# Large deformation diffeomorphic mapping of 3D shape variation reveals two distinct mandible and head capsule morphs in *Atta vollenweideri* leaf‐cutter worker ants

**DOI:** 10.1002/ece3.11236

**Published:** 2024-04-16

**Authors:** Natalie Imirzian, Frederik Püffel, Flavio Roces, David Labonte

**Affiliations:** ^1^ Department of Bioengineering Imperial College London London UK; ^2^ Department of Behavioural Physiology and Sociobiology Biocenter, University of Würzburg Würzburg Germany

**Keywords:** allometry, ants, morphology, morphometrics, polymorphism, social insects

## Abstract

Ants are crucial ecosystem engineers, and their ecological success is facilitated by a division of labour among sterile “workers”. In some ant lineages, workers have undergone further morphological differentiation, resulting in differences in body size, shape, or both. Distinguishing between changes in size and shape is not trivial. Traditional approaches based on allometry reduce complex 3D shapes into simple linear, areal, or volume metrics; modern approaches using geometric morphometrics typically rely on landmarks, introducing observer bias and a trade‐off between effort and accuracy. Here, we use a landmark‐free method based on large deformation diffeomorphic metric mapping (LDDMM) to assess the co‐variation of size and 3D shape in the mandibles and head capsules of *Atta vollenweideri* leaf‐cutter ants, a species exhibiting extreme worker size‐variation. Body mass varied by more than two orders of magnitude, but a shape atlas created via LDDMM on μ‐CT‐derived 3D mesh files revealed only two distinct head capsule and mandibles shapes—one for the minims (body mass < 1 mg) and one for all other workers. We discuss the functional significance of the identified 3D shape variation, and its implications for the evolution of extreme polymorphism in *Atta*.

## Graphical abstract

*Atta vollenweideri* leaf‐cutter ant workers vary continously in size across more than two orders of magnitude in body mass. We investigated whether size‐variation correlates with shape variation by analysing the 3D morphology of mandibles and head capsules. We identify two distinct worker morph, and link these shape differences to functional specialisation.

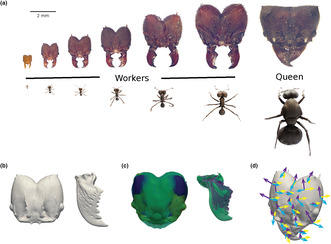

## 1 INTRODUCTION

Social insects, such as ants, termites, and some bees and wasps, dominate terrestrial ecosystems (Schultheiss et al., 2022; Wilson, 1990). An important factor contributing to their ecological success was the evolution of a non‐reproductive worker caste, allowing task specialisation and parallelisation with limited reproductive conflict (Bernadou et al., 2021; Oster & Wilson, 1978). This division of reproductive labour also enabled morphological differentiation between the sterile and reproductive castes that likely revolutionised the colony economy (Peeters & Ito, 2015). For example, in ants, sterile females (workers) lost wings, flight musculature, and often ocelli (Kronauer, 2020). The thorax, freed from the demands imposed by the need to fly, instead evolved into a ground labour “power core” which holds large muscles for neck stabilisation and legged locomotion, both crucial adaptations for central place foraging (Peeters et al., 2020).

In about 15%–20% of all ant species, caste differentiation transcends this ancient division into reproductive and sterile castes, and further morphological variation exists within the sterile caste itself (Oster & Wilson, 1978; Wilson, 1963). For example, turtle ants (*Cephalotes* spp.) possess large sterile morphs with a head shape that evolved to function as a physical block to the colony entrance (Corn, 1980; Powell, 2008); and *Eciton* army ants have large morphs with extremely enlarged mandibles, specialised for piercing (Powell & Franks, 2006). Both examples illustrate that social insects with morphological worker diversity also tend to exhibit morphological polyethism (Beshers & Fewell, 2001): *Cephalotes* and *Eciton* workers with different morphology perform distinct tasks, and may well be distinguishable on the basis of behaviour alone.

A correlation between worker morphology and task preference is typically thought to reflect advanced task specialisation, favoured because it increases colony fitness (Oster & Wilson, 1978, but also see Cole, 2020 for a recent criticism of this suggestion). Indeed, classic ergonomic theory suggests that ant colonies maximised their fitness if each essential task was performed by a morphologically specialised worker (Oster & Wilson, 1978). However, this prediction stands in sharp contrast to empirical observations: ant workers may perform more than 40 tasks (Wilson, 1976), but no more than four to five “worker castes” have been reported for any ant species (Franks, 1985; Muratore et al., 2023; Silva et al., 2016). One possible explanation for this seeming discrepancy may be that task specialisation can be achieved with isometric variations in body size alone, with the putative advantage that no complex changes in the developmental programme of workers are needed.

An extreme example of worker size variation can be found in the leaf‐cutter ants (Subtribe Attina): workers may fall anywhere on a body mass continuum spanning up to two orders of magnitude (Schultz & Meier, 1995; Weber, 1972). Famed for their fungal agriculture system, leaf‐cutter ant workers cut leaves at an industrial rate to maintain a fungal crop, which serves as food for the colony. Larger leaf‐cutter ants possess relatively more muscle mass and thus can generate larger bite forces (Püffel et al., 2021), providing the colony with access to a larger range of plant material (Püffel, Roces, et al., 2023). Indeed, larger workers are more likely to cut tougher leaves (Clark, 2006; Evison & Ratnieks, 2007; Nichols‐Orians, 1991), illustrating how size‐differences can be functionally advantageous. However, whether, and if so how, leaf‐cutter ant workers also differ in shape is controversial; the number of morphs reported in the literature lies anywhere between one and five (Cherrett, 1972; Feener et al., 1988; Franks & Norris, 1987; Hernandez & Caetano, 1995; Muratore et al., 2023; Rudolph & Loudon, 1986; Silva et al., 2016; Wetterer, 1991, 1999). The key difficulty is that shape variation can be subtle, and is thus generally harder to quantify than differences in body size. Distinguishing between differences in body size and shape is important for at least two reasons. First, it has direct implications for the explanatory power of ergonomic theory and our understanding of the functional significance of continuous polymorphism in the leaf‐cutters (Oster & Wilson, 1978; Wilson, 1980a, 1980b, 1983); and second, it may reveal the extent to which developmental constraints limit morphological variation within workers (e.g. Franks & Norris, 1987; Pie & Traniello, 2007; Tschinkel, 2013), and so improve our understanding of the evolution of worker polymorphism in general.

The classic approach to separating size and shape is allometric analysis, i.e. the systematic study of co‐variations of characteristic body dimensions with animal size (Gould, 1966; Huxley, 1924; Schmidt‐Nielsen, 1984). Allometric studies have been used to great success to study shape variation in ant workers, including leaf‐cutters (Feener et al., 1988; Rajakumar et al., 2018; Tawdros et al., 2020; Wetterer, 1991; Wills et al., 2018; Wilson, 1953). However, the key advantage of allometry – the required morphological measurements are often straightforward – is also its main weakness: allometric analyses are typically concerned with the covariation of characteristic distances, areas or volumes. In other words, complex 3D shapes are reduced to simple linear, areal or volumetric descriptors defined a priori by the observer, limiting the quantitative prowess and introducing potential for observer bias. A powerful alternative to allometric studies is quantitative 3D shape analysis, a formidable task, rendered both possible and feasible by major advances in geometric morphometrics in the past three decades (Adams et al., 2013; Klingenberg, 2010; Mitteroecker et al., 2013; Rohlf, 1990; Rohlf & Marcus, 1993).

Geometric morphometrics overcomes some limitations of allometry by describing shape variation via the relation between the Cartesian coordinates of key points; they are the conceptual offspring of D'Arcy Thompson's work on growth and form (Thompson, 1917). The dominant methodological approach relies on landmarks – carefully chosen points of equivalency that can be identified without ambiguity across different objects (Bookstein, 1991; Rohlf & Slice, 1990). Landmarks enable powerful and meaningful statistical comparisons, but also retain some of the limitations of allometric analyses: landmark placement is observer‐dependent (Percival et al., 2014; Shearer et al., 2017; von Cramon‐Taubadel et al., 2007), and the complexity of the shape changes that can be identified is directly linked to the number of landmarks, so inducing an effort‐accuracy trade‐off (e.g. Toussaint et al., 2021). In some cases, such as large, smooth surfaces, it may be impossible to reliably place landmarks across different specimens altogether. State‐of‐the art methods that employ sliding and surface semilandmarks are one way to circumvent this issue, as they can be applied even to curved and smooth surfaces, and typically outperform landmark analyses; they do, however, remain time consuming (Bardua et al., 2019; Goswami et al., 2019). Accordingly, there has been significant interest in developing either methods for automated landmark placement, or techniques that do not rely on landmarks to begin with (Aneja et al., 2015; Boehm et al., 2011; Braga et al., 2019; Durrleman et al., 2014; Koehl & Hass, 2015; Percival et al., 2019; Pomidor et al., 2016). Landmark‐free approaches are particularly attractive, because they remove some of the residual disadvantages of allometry that landmark‐based approaches retain. They (1) reduce potential for bias; (2) enable analysis with higher spatial resolution (placing landmarks is often a time bottleneck); and thus (3) render work with large 3D datasets feasible.

Surface matching methods are one option for landmark‐free shape analysis (e.g. Boyer et al., 2011; Durrleman et al., 2014; Koehl & Hass, 2015). Unlike the use of landmarks and semi‐landmarks, where the user must specify areas of interest a priori, surface matching requires no such choice, and is thus more objective. Additionally, surface matching approaches can efficiently and accurately capture the shape of non‐uniform curved regions, such as cusps, basins, and grooves; landmark‐based approaches for these regions require careful placement of many semi‐landmarks. In diffeomorphic surface matching (see Durrleman et al., 2012, 2014), shapes can be represented by continuous surfaces instead of a limited number of homologous points, and point correspondence between shapes is not required. Shape differences are characterised by deformations, instead of point positions, allowing for accurate quantification of even minute shape differences that cannot be detected with landmark (Toussaint et al., 2021) or semi‐landmark (Braga et al., 2019) based methods.

In this work, we use landmark‐free morphometrics in the form of large deformation diffeomorphic metric mapping to investigate the 3D variation of shape in extremely polymorphic *Atta vollenweideri* (Forel 1893) leaf‐cutter ants. As the cutting and transport of leaves is of elemental importance to leaf‐cutter ant ecology, and because head capsules are often a region of great morphological specialisation in ants (Booher et al., 2021; Pie & Traniello, 2007; Powell et al., 2020) we focus on the shape variation of head capsules and mandibles in all morphs from the smallest workers to the queen. We aim to (i) identify systematic variations in shape to determine the number of structurally discrete head and mandible morphs; (ii) link shape variations with task preferences to discuss their functional significance; and (iii) briefly place our findings in the broader context of the evolution of worker polymorphism in ants.

## 2 METHODS

### 2.1 Study organism and sample preparation

We sampled 20 ant workers and one queen from a single *Atta vollenweideri* colony, collected in Uruguay in 2014, and housed in multiple plastic boxes connected by plastic tubing (inner diameter = 25 mm), with separate boxes for the fungus, waste, and plant material provided as food. Based on the fungal volume, we estimate the colony contained approximately 300,000 individuals. The colony was fed bramble leaves (*Rubus* spp.) five times a week, supplemented with corn flakes and honey water ad libitum. Twenty ant workers, across the approximate total size range from 0.3 to 43.3 mg, were removed from the foraging area, and weighed to the nearest 0.1 mg immediately after removal (Explorer Analytical EX124, max. 120 g × 0.1 mg, OHAUS Corp., Parsippany, NJ, USA). Ants were selected so that the spacing in mass was approximately equal in logarithmic space. The sample size was limited by the time‐intensive post‐processing and segmentation of CT scans (see below), but is comparable to or larger than sample sizes in related recent work (Aibekova et al., 2023; Casadei‐Ferreira et al., 2021; Hita‐Garcia et al., 2019; Klunk et al., 2021).

To isolate the heads and mandibles from the main body, ants were sacrificed by freezing, and subsequently decapitated using a razor blade. The antennae were removed as they had inconsistent orientation, impeding alignment of the scans, and are not the focus of this study. All heads were fixed in paraformaldehyde for 18 h (4% in PBS, Thermo Fisher Scientific, Waltham, MA, USA), and then stored in 100% ethanol. The queen head was not fixed, but stored in a −18°C freezer in 100% ethanol; queen weight was determined only after ethanol storage. Both differences arose because we collected the queen from the laboratory colony after she had died naturally, and the exact time of her death was uncertain. Ethanol storage likely leads to an underestimate of wet mass (Knapp, 2012), and the measured weight of 256.5 mg is lower than what has been previously recorded as the mass of an *A. vollenweideri* queen (Vieira et al., 2015). The queen mass is not used for any inference, and we thus did not implement any further correction to the ethanol wet mass. Throughout the manuscript, we follow the morphological terminology from (Richter et al., 2020, 2021, 2022).

### 2.2 Computed tomography and scan post‐processing

**FIGURE 1:**
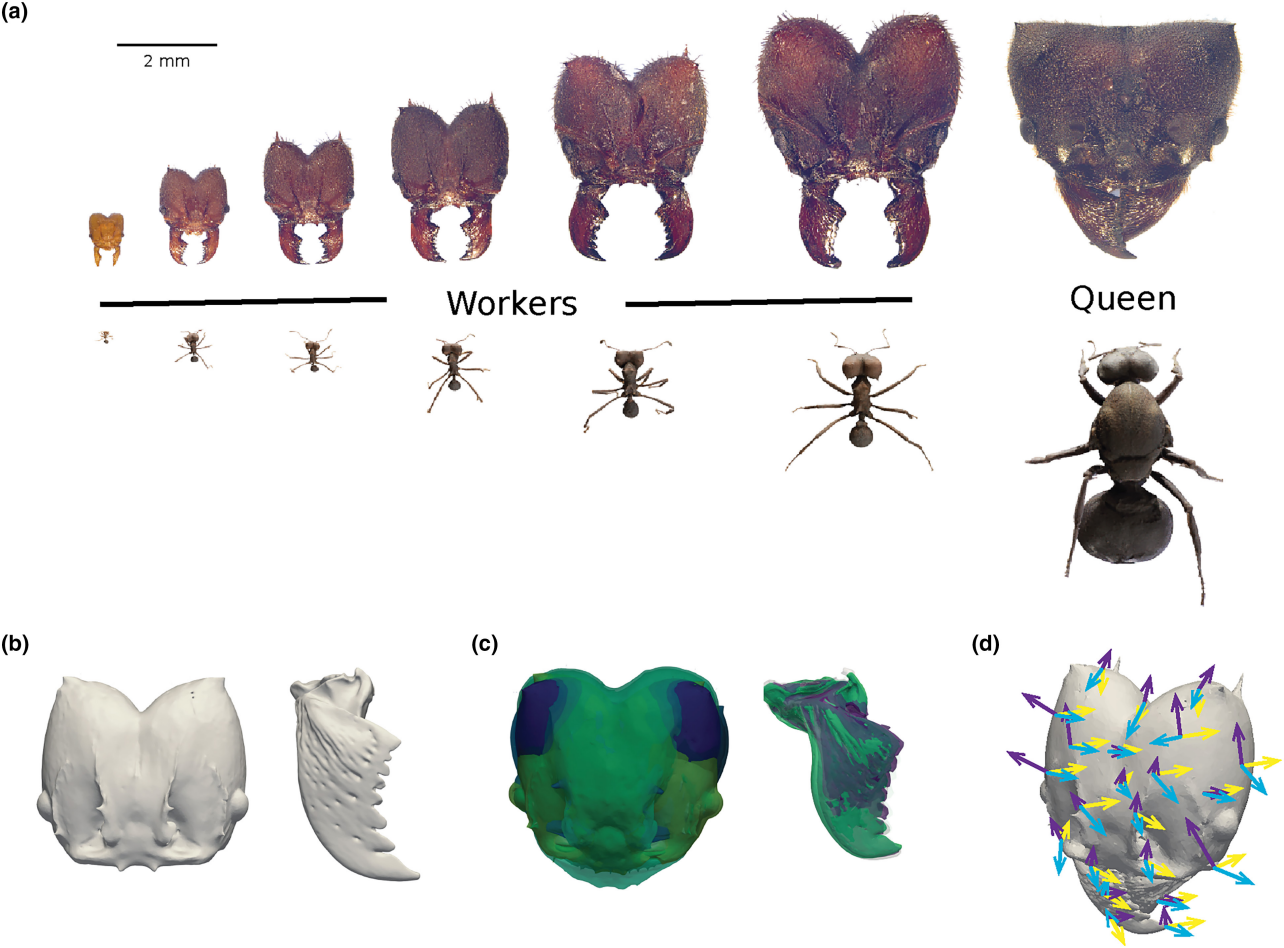
(a) *Atta* leaf‐cutter ants are highly polymorphic. Sterile workers may vary in body mass by more than two orders of magnitude (top view of whole‐body 3D models of *Atta vollenweideri* generated with scAnt, Plum & Labonte, 2021). We investigated if workers differ not only in size, but also in shape, focussing on the head capsule and mandibles as illustrated by macrophotographs – two tagma of key functional importance in the leaf‐cutters. To this end, we (b) cleaned and decimated segmented micro‐CT scans of 20 head capsules and mandibles to generate mesh files, which were (c) subsequently aligned via Procrustes transformation to remove differences in size and orientation; colours indicate different meshfiles. (d) 3D shape variation was then assessed via landmark‐free Large Deformation Diffeomorphic Metric Mapping. A template complex shape was estimated by placing control points in areas of large shape variation. The deformation required to warp the template complex into individual shape complex is calculated through minimisation of a cost function, and parameterised by “momenta”, vectors (arrows) assigned to each control point (for more details see Section 2).

Micro‐computed tomography (μCT) scans were conducted and processed according to the procedure described in detail by Püffel et al. (2021), apart for the queen, which was scanned at a later date with a different scanner (Appendix S1). Threshold‐based segmentation of the head capsules and mandibles was performed in ITK‐SNAP (v. 3.6; Yushkevich et al., 2006; Figure 1b), using the image stacks obtained from the μCT scans. After segmentation, the head capsule and right mandible were extracted as distinct mesh files with triangular surface elements via the native VTK function in Python (v.3.8). The right mandible was selected for further analysis. We did not expect morphological differences between left and right mandible because leaf‐cutter ants have bilaterally symmetric heads (Püffel et al., 2021), and are ambidexterous, with only weak preferences for one mandible over the other (Jasmin & Devaux, 2015).

Each extracted mesh file had more than 1,000,000 vertices. In order to reduce their density, each mesh was decimated to 0.1% or 1% of its original size using the Quadric Edge Collapse Decimation function in MeshLab (v. 2020.03; Cignoni et al., 2008). Decimation percentage was selected as the largest reduction that preserved all relevant anatomical features, assessed via visual inspection. Following decimation, minor errors, such as small holes or voxels from other body parts, were corrected manually within MeshLab and Blender (v. 2.9); we note that the shape analysis method used here is in principle robust to noise, mesh errors, and inconsistencies in mesh orientation (see Durrleman et al., 2014). Mesh quality was improved further by removing duplicated faces, and faces from non‐manifold edges, followed by Laplacian smoothing with 2‐time steps in MeshLab. Mesh files were inspected to ensure smoothing did not alter projections on the mesh surface, and that all morphological features were retained. The decimated and cleaned mesh files were exported as VTK files (Figure 1b). Representative mesh files across the size range are available on SketchFab (https://skfb.ly/oFPAM).

### 2.3 Large deformation diffeomorphic metric mapping

D'Arcy Thompson proposed to quantify shape differences via the deformation of the ambient space that is required to “warp” one shape into another (Thompson, 1917). One modern technical implementation of this idea is large deformation diffeomorphic metric mapping (LDDMM). We use a specific instance of this approach, based on control points (Durrleman et al., 2014), implemented in the software Deformetrica (Bône et al., 2018). We chose this particular implementation because it performed as well or better than traditional landmark‐based approaches at identifying cranial dysmorphologies in mice (Toussaint et al., 2021).

Shape analysis with Deformetrica consists of two main steps: the estimation of a “template complex”, which represents the mean shape across all subject shape complexes; and the computation of the deformation required to warp the template complex into each subject shape complex (referred to as shape registration; Bône et al., 2018; Durrleman et al., 2014). The key idea enabling this shape analysis is to parameterise the ambient space via a set of control points, each with an associated vector which encodes deformation. This simple parameterisation has the advantage that it can encode a large variety of ambient space transformations, and thus solves optimisation problems with reasonable computational effort (Durrleman et al., 2014). The combination of a template complex and the set of deformations for each subject complex defines a “shape atlas”.

To create the shape atlas, we first scaled and aligned all input meshes. To this end, five landmarks were placed on the mandibles, and nine landmarks on the head capsules, using MITK (v. 2021.02; Wolf et al., 2004). Landmark locations are provided in Appendix S1; preliminary trials suggested that this number of landmarks was sufficient to ensure alignment. Subsequently, a Procrustes transformation was performed using VTK's Procrustes alignment function in Python. The centroid values were examined, and the meshes visually inspected to ensure successful alignment (Figure 1c).

Next, we computed the template shape and the deformations required to optimally warp the template complex into each subject complex. which requires specification of two hyperparameters: the Gaussian kernel width and noise (see Bône et al., 2018). The kernel width influences the number of control points, which are in essence unbiased landmarks. Consequently, a large kernel width results in smooth global deformations, and a small kernel width captures small‐scale deformations, but may result in uneven surfaces. The kernel width was systematically altered in initial trial‐and‐error test runs, and we visually inspected the output to select a combination that led to visually consistent and undistorted morphing (see also Toussaint et al., 2021). The kernel width was set to 0.25 for the mandibles and the head capsules, resulting in 315 and 1890 control points, respectively; the difference in number reflects the difference in relative size of the objects. The noise parameter was set at 0.1 for both body parts, and the number of intermediate warped shapes between the template and the individual shape was set to 20. Optimisation was performed through a steepest gradient descent optimisation procedure on the cost function (Bône et al., 2018), with an initial optimisation step size of 0.1, and a maximum of 500 and 2000 iterations for the mandibles and head capsules, respectively. Preliminary trials suggested that log‐likelihood levelled out after 500–1000 iterations for both body parts.

The final result of this process is a shape atlas for both head capsules and mandibles: an average 3D mesh which represents the population shape; a set of control points concentrated in areas of high variability; and “momenta” for each control point which describe the directional deviation from the template complex for each subject complex.

### 2.4 Allometry of cuticle thickness and mandible second moment of area

Through the shape atlas, we are able to quantify differences in external head capsule and mandible shape. However, internal shape parameters may also vary with size. We characterised internal shape by extracting two metrics relevant to the mechanical demands induced by biting: the thickness of the cuticle of both head capsule and mandible, which influences cuticle deformation (Püffel, Meyer, et al., 2023); and the second moment of area of the mandible, which determines its rigidity in bending (see e.g. Kundanati et al., 2020). Both parameters were extracted from the segmented image stacks, i.e. prior to Procrustes transformation, using Fiji (v. 2.2; Schindelin et al., 2012).

In order to extract cuticle thickness, we used the *Analyse Local Thickness* function implemented in BoneJ (Doube et al., 2010), which extracts the “true” local thickness across the entire 3D mesh: small seeds are grown into spheres inside the segmented tissue until they reach the tissue boundary; the local thickness is then extracted as the shortest distance between two boundaries. Tests with simple shell‐like model geometries confirmed that this method has an error of less than 1%, and yields results independent of image stack orientation.

The second moment of area describes the distribution of mass around a reference axis, and is of relevance in the context of ant mandibles because it determines their resistance to bending deformation. The bending stresses induced by biting are situated in the mandible cross‐sections spanned by the rotational axis and bite force vector. In order to extract the second moment of area about the rotational axis, we first oriented the image stacks such that the slices were perpendicular to the mandible main axis, defined as the line connecting the joint centre with the tip of the most distal tooth (Püffel et al., 2021), using the ImageJ plug‐in transformJ (Meijering et al., 2001); this anatomical axis is approximately perpendicular to both bite force vector and rotational axis (Püffel et al., 2021). Next, we used the *Orientation* function native to BoneJ to align the principal axis of the image cross‐sections such that it was roughly perpendicular to the serrations of the mandible masticatory margin, and thus approximately parallel to the rotational axis. We then extracted the second moment of area for each image cross‐section along the mandible main axis, and calculated the average for each mandible, with the dimension of length to the fourth power.

### 2.5 Statistical analyses

The output of the statistical shape model is a mean or template shape, alongside a set of control points and momenta that indicate the deformation required to warp the template into each sample shape. The direction of movement is indicated at each control point by a 3‐dimensional vector, known as the momentum, with the paired set of control points and vectors defining a vector “velocity” field of movement. The sum of the magnitude of all momenta per sample, from here on referred to as the total distance to the mean shape (TdtMS), thus serves as a suitable proxy for the magnitude of shape differences. To investigate whether head capsules and mandibles fall into distinct clusters or “shape groups”, for example with respect to ant size, we performed a principal component analysis (PCA) on the momenta (3D vectors, consisting of *x*, *y*, and *z* components) using the scikit‐learn package in Python (v. 3.7.7; Pedregosa et al., 2011). The number of components was selected such that 95% of the total variance was explained. Prior to the PCA, the data were scaled to fall between 0 and 1, by subtracting by the minimum value and dividing by the range. We chose min‐max scaling as it preserves the original shape of the distribution, and does not alter the importance of outliers in the data (Pedregosa et al., 2011). Indeed, many momenta are “inliers” – they have a value close to zero, and it is thus reasonable to compress these values and expand the differences between larger values.

To investigate the allometry of cuticle thickness and mandible second moment of area, we performed an ordinary least squares regression on log10 transformed data using the stasmodels package in Python (Seabold & Perktold, 2010). The regression estimated the coefficients *a* and *b* in the scaling equation *y* = *aM* ^*b*^ as log_10_(*y*) = log_10_(*a*) + *b* × log_10_(*M*), where *M* is the mass in milligrams, *y* is the thickness or the second moment of area, and *b* is the scaling coefficient. We tested if the relationship departs from the null hypothesis (*H*_0_) of isometry, which predicts a slope of *b =* 1/3 for thickness and a slope of *b* = 4/3 for the second moment of area. The queen was excluded from all allometric analyses because her live body mass was unknown.

## 3 RESULTS

### 3.1 Both head capsule and mandible shape vary across morphs

**FIGURE 2:**
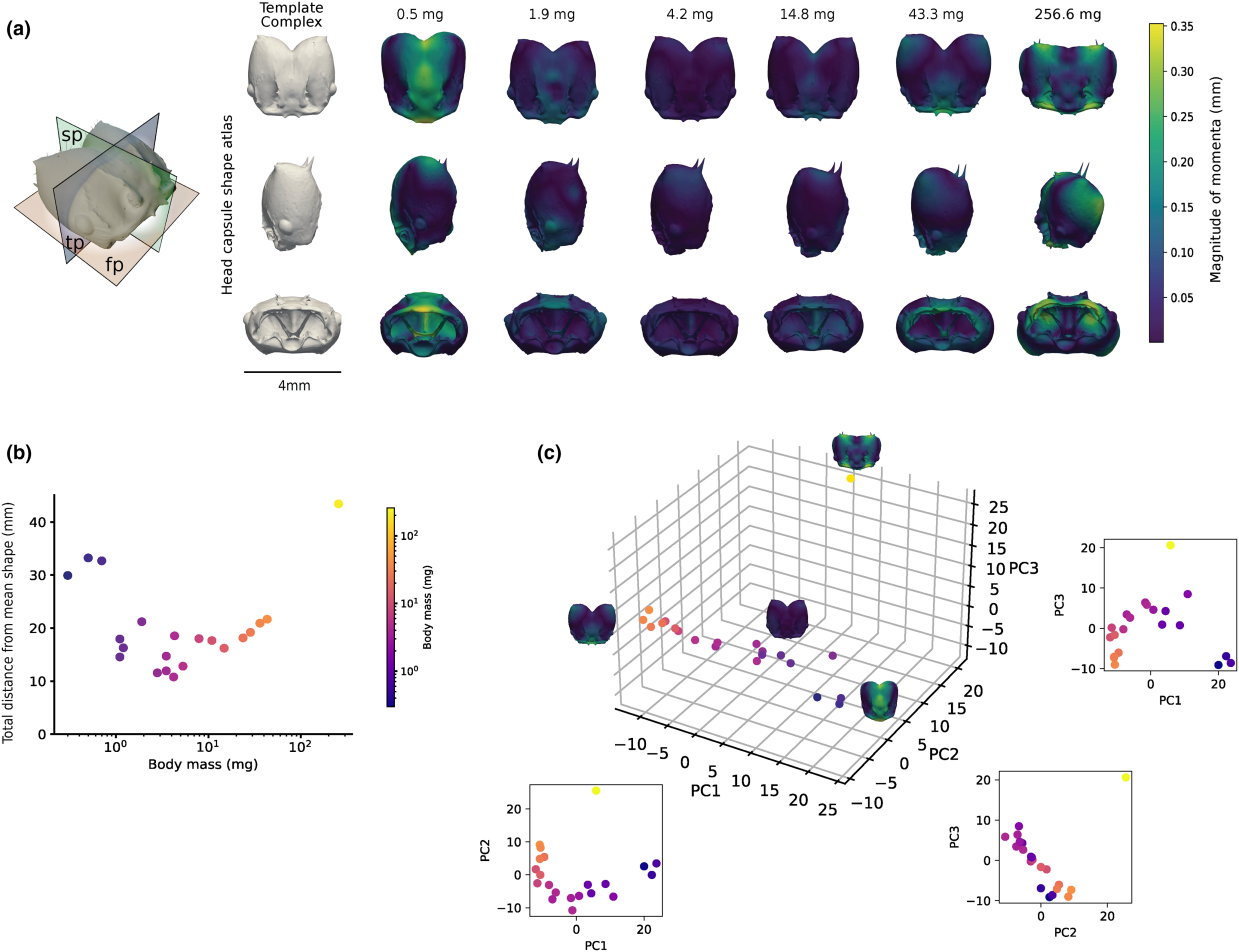
Head capsules vary not only in size but also in shape in *Atta vollenweideri* leaf‐cutter ants. (a) Creation of a shape atlas revealed that head capsules shift from an elongated barrel‐shape to a heart‐shape between minims (body mass < 1 mg) and medias (all other workers); the queen head capsule has a distinct more rectangular shape. Colours on the individual 3D mesh files decode the distance from the template complex, from dark blue (no change) to green (medium change) to bright yellow (maximum change). The top images show the dorsal view along the frontal plane (fp), the middle images show the lateral view along the sagittal plane (sp), and the bottom images show the anterior view along the transverse plane (tp). The (b) Total distance to Mean Shape (TdtMS), the sum of the magnitude of all momenta, serves as a proxy for the extent of shape change between template and each individual shape. The TdtMS is largest for minims and the queen, and intermediate and of approximately equal magnitude for medias. (c) A principal component analysis on the momenta confirms an approximate grouping of shapes into minims, medias, and the queen.

**FIGURE 3:**
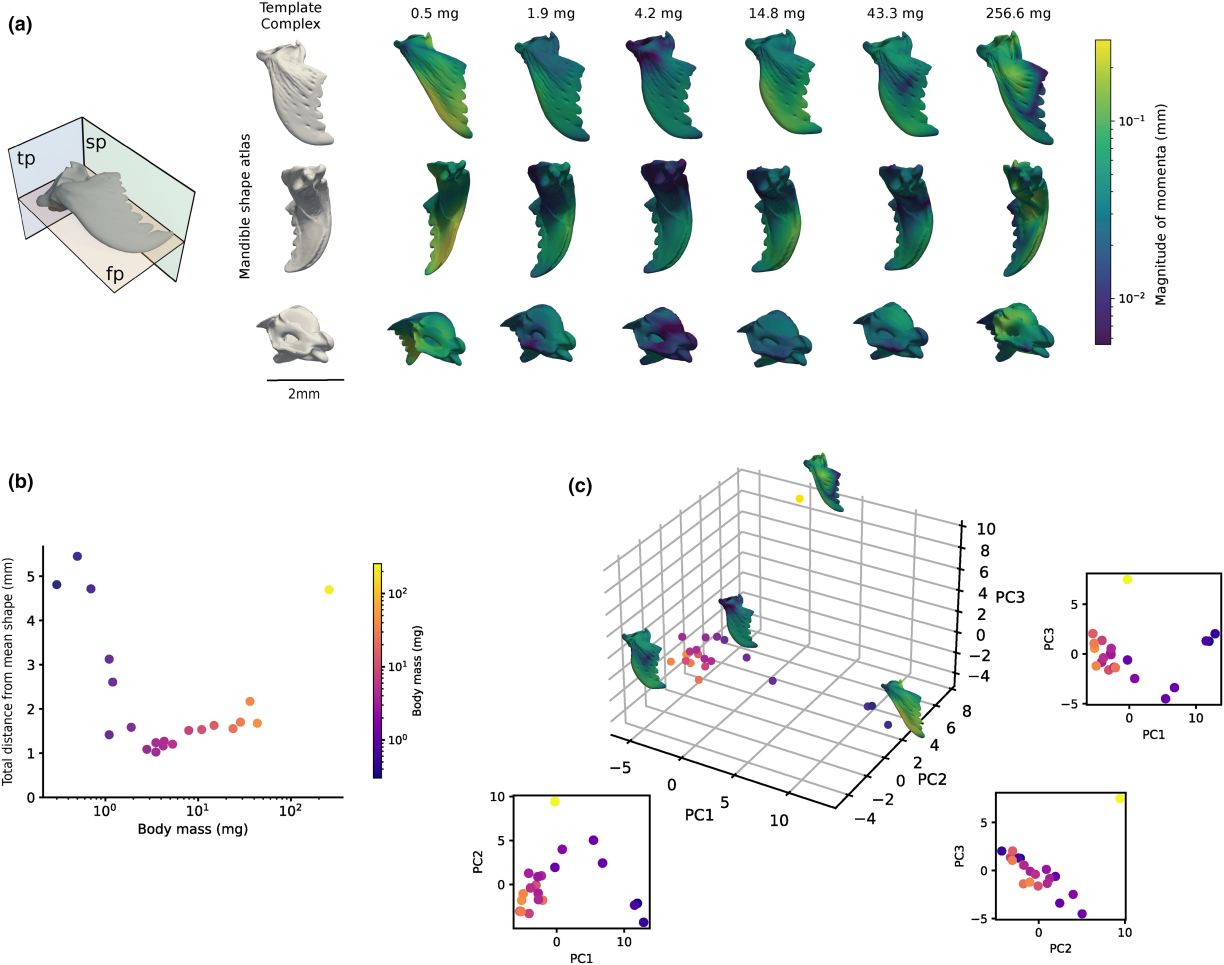
Mandibles vary not only in size but also in shape in *Atta vollenweideri* leaf‐cutter ants. (a) Creation of a shape atlas revealed that mandibles of minims (body mass < 1 mg) have triangulate blades, and a narrow basal stem. In medias (body mass > 1 mg), the mandible blade warps into a sickel‐like shape, driven by an increase in curvature, and an enlargement and re‐orientation of the apical tooth. The queen mandible combines morphological elements of minim and media mandibles. Colours on the individual 3D mesh files decode the distance from the template shape, from dark blue (no change) to green (medium change) to bright yellow (maximum change). The top images show the dorsal view along the frontal plane (fp), the middle images show the lateral view along the sagittal plane (sp), and the bottom images show the posterior view along the transverse plane (tp). (b) The sum of all momenta, the Total distance to Mean Shape (TdtMS), is a proxy for the total magnitude of shape change required to warp the template complex into the individual shapes. It is largest for minims and the queen, and of intermediate and approximately equal magnitude for medias. (c) A principal component analysis on the momenta confirms an approximate grouping of shapes into minims, medias and the queen.

The shape atlas revealed pronounced differences in both head capsule and mandible shape across morphs (Figures 2 and 3). The total distance to the mean shape (TdtMS) was largest for the smallest workers (approximately <1 mg) and the queen; it was of smaller and similar total magnitude for all other workers (Figures 2a and 3a). Both heads and mandibles of these three groups indeed appear morphologically distinct to the eye (Figure 1a), suggesting the existence of three structurally distinct head capsule and mandible morphs in *A. vollenweideri* colonies: (i) small workers with a body mass less than approximately 1 mg (from here on *minims*); (ii) all larger workers (from here on *medias*); and (iii) the queen.

### 3.2 Worker head capsules are barrel or heart shaped

Relative to the template shape, the head capsules of minims are elongated along their anterior–posterior axis, and narrowed along the lateral axis, resulting in a ‘barrel‐shaped’‐appearance in the horizontal plane. Most of this shape variation is concentrated along the central line of bilateral symmetry, i.e. in the sagittal plane (see Video 1, Figure 2a). The head capsule of all medias, in turn, is characterised by an outward expansion of the posterior lobes, resulting in an increased lobe curvature, and thus a “heart‐shaped”‐appearance in the horizontal plane. Shape differences are less pronounced in the lateral view, where the head capsules of all workers appear approximately elliptical, with a major anterior–posterior and minor dorso‐ventral axis (Figure 2a; Video 1).

**VIDEO 1:** The output from the shape atlas provides a template shape and the deformation required to morph from the template shape into each sample shape. The video shows the morphing of the 3D mesh respresentation of the template head capsule to the individual head capsules for four sizes of ants: workers sized 0.5, 4.2, and 43.3 mg, and the queen. The colouration on the surface of the mesh indicates the resulting distance from the template shape, with the colour scale indicated in the right corner in millimeters. Videos were created in ParaView (Ahrens et al., 2005).

The head capsule of the queen has a shape distinctly different from both minims and medias: the posterior section of the head is dorso‐ventrally flattened, and the head capsule is approximately symmetric in the transverse plane, due to a combination of lateral widening and anterior–posterior shortening. In lateral view, it is almost round. Most modifications of the queen head relative to the template shape are concentrated around the mandible articulation anteriorly, and the two characteristic head spikes posteriorly (Figure 2a; Video 1).

The qualitative clustering into three morphs is confirmed both by inspection of the TDtMS, and by principal component analysis (Figure 2b,c). The TDtMS separated morphs into three groups; one containing all minims; one containing only the queen – both characterised by a large TDtMS; and one including the broad intermediate size range from about 2 to 45 mg, which all had a similar TDtMS of smaller magnitude (Figure 2b). Because the same TDtMS can correspond to different actualised shapes, we inspected the results of a principal component analysis on the momenta to confirm the clustering, and to study shape variation within the medias further.

A total of 14 components were required to explain 95.4% of the variation in head capsule shape, highlighting the complexity of the realised 3D shape variation; the first three components explained 35.7%, 16.9%, and 14.1%, or a total of 66.8% of the variance, respectively (Figure 3). Minims, medias, and the queen occupied distinct positions in shape space; the PCA also revealed that head capsule shape varies systematically along PC 1 for medias that had a similar intermediate TdtMS. Indeed, PC1 scores correlated strongly with body mass within medias (Figure S2A; Pearson's correlation = −0.63; *p* = .01), and appear to primarily encode variation in the curvature and relative size of the posterior lobes (Figure 2c). The queen head capsule shape is primarily distinguished by a substantially different score for PC 2 and PC 3, which appear to reflect changes in the head capsule aspect ratio in the transverse plane and in regions close to mandible articulation, as well as anterior–posterior shortening of the head capsule – all of which varied little across the workers (Figure 2a – anterior view & Figure 2c).

### 3.3 Mandibles are triangulate or sickle‐shaped

Mandible shape differed considerably across morphs (Figure 3). In minims, the masticatory, lateral and basal margins of the mandible, which together define the shape of the mandible blade, are approximately straight (Figure 3a; Video 2). The mandible blades of minims thus appear triangular, a likely morphological synapomorphy of the poneroid‐formicoid lineage (Richter et al., 2020, 2022). The basal margin of minim mandibles is elongated, seemingly at the expense of the basal stem, which is the location of the ventral and dorsal mandibular articulation, the trulleum, and of the atala (or “abductor swelling” Richter et al., 2019, 2020). The basal stem is relatively narrower in minim compared to media mandibles. The apical incisor of minim mandibles is only marginally larger than more proximal tooth‐like serrations, and forms shallow angles with respect to the masticatory margin (Figure 3a – dorsal view).

**VIDEO 2:** The output from the shape atlas provides a template shape and the deformation required to morph from the template shape into each sample shape. The video shows the morphing of the 3D mesh representation of the template mandible to the individual mandibles for the fours sizes of ants: workers sized 0.5, 4.2, 43.3 mg, and the queen. The colouration on the surface of the mesh indicates the resulting distance from the template shape, with the colour scale indicated in the right corner in millimeters. Videos were created in ParaView (Ahrens et al., 2005).

In medias, the basal margin remains straight but is relatively shorter, the basal stem is wider, the masticatory and lateral margins curve inward, the apical incisor increases in relative length, and its orientation changes continuously such that it was almost perpendicular to the masticatory margin in the largest workers (Figure 3a; Video 2). The increase in curvature appears most strongly pronounced for the lateral margin, at least partially as a result of the increase in length and change in orientation of the apical incisor. In combination with a concavity of the masticatory margin, as seen in dorsal view, these changes result in a sickle‐like shape of the mandibular blade in medias, reported also in other leaf‐cutter ant species (Figure 3a; Video 2 and Hernandez & Caetano, 1995; Keller, 2011; Mayhe‐Nunes & Caetano, 1994; Silva et al., 2016).

The queen mandible, in turn, is characterised by a mandibular articulation and basal margin of intermediate width and length, and an intermediate orientation and reduced elongation of the apical incisor. As a result, the curvature of the lateral and masticatory margins appears more moderate (Figure 3a); the queen mandible seems to combine some morphological characteristics of both minim and media mandibles.

All mandibles exhibited a dorsal twist toward the internal margin, a characteristic morphological feature of the mandibles of most female ants regardless of shape variation in the mandible blade itself (Boudinot et al., 2021; Keller, 2011). However, this “torsion” appeared slightly less pronounced in the smallest workers (Figure 3a). Neither the dorsal or ventral mandibular articulations nor the atala or the trulleum appear to vary much in shape across workers, though they are relatively smaller in minims (Figure 3a – posterior view and Video 2). The queen atala shows slight dorsoventrally widening and a deeper and narrower trulleum; the grooves on the ventral side of the mandibular stem also appear deeper.

The clustering into three distinct mandible shape categories is confirmed both by the TDtMS and by PCA (Figure 3b,c). Warping the template complex into minim and queen mandibles required a TDtMS about a factor of three to four larger than for medias, which were all characterised by a similar TDtMS (Figure 3b). Principal component analysis confirmed this clustering and allowed us to inspect shape variations within media mandibles (Figure 3c). A total of 10 components were required to explain 95% of the variation; the first three components explained 52.4%, 15.5%, and 8.6% or 76.6% of the total variance, respectively. Minims and medias fell in distinct clusters along PC1, with the queen grouping closer to smaller media workers (Figure 3c). PC1 appears to encode the relative size and orientation of the apical incisor, and the curvature of the lateral margin. Larger workers vary slightly but systematically along PC 2 (Figure S2; Pearson's correlation = 0.59; *p* = .005), which seemingly encodes the relative size of the basal margin and the mandibular articulations. The queen mandible varied largely on PC3, likely reflecting a lateral shift in the dorsal ridge (yellow area in Figure 3a – dorsal view), or changes in the mandibular stem such as deeper ventral groves and a dorsoventrally wider atala (Figure 3a – posterior/lateral view). The queen mandible occupied distinct PC2 and PC3 coordinates, but scored a PC1 comparable to that of intermediate‐sized worker mandibles (Figure 3c).

### 3.4 Co‐variation of mandible and head shape

The first principal components, reflecting curvature and posterior lobe size for the head capsules, and changes in the apical incisor and curvature of the lateral margin for the mandibles, were positively correlated (Figure S2; Pearson's correlation = .92; *p* = 2.66 × 10^−9^). In contrast, the second principal components, reflecting head capsule aspect ratio and changes in mandible articulation for the head capsules, and alterations in the basal margin and mandibular articulation for the mandibles, did not show a significant correlation (Figure S2; Pearson's correlation = .23; *p* = .31). However, the weak correlation is dominantly caused by the queen, which is an outlier, and the correlation increases in strength and becomes significant when this point is removed (Pearson's correlation = −.50; *p* = .02).

### 3.5 Allometry of cuticle thickness and second moment of area

**FIGURE 4:**
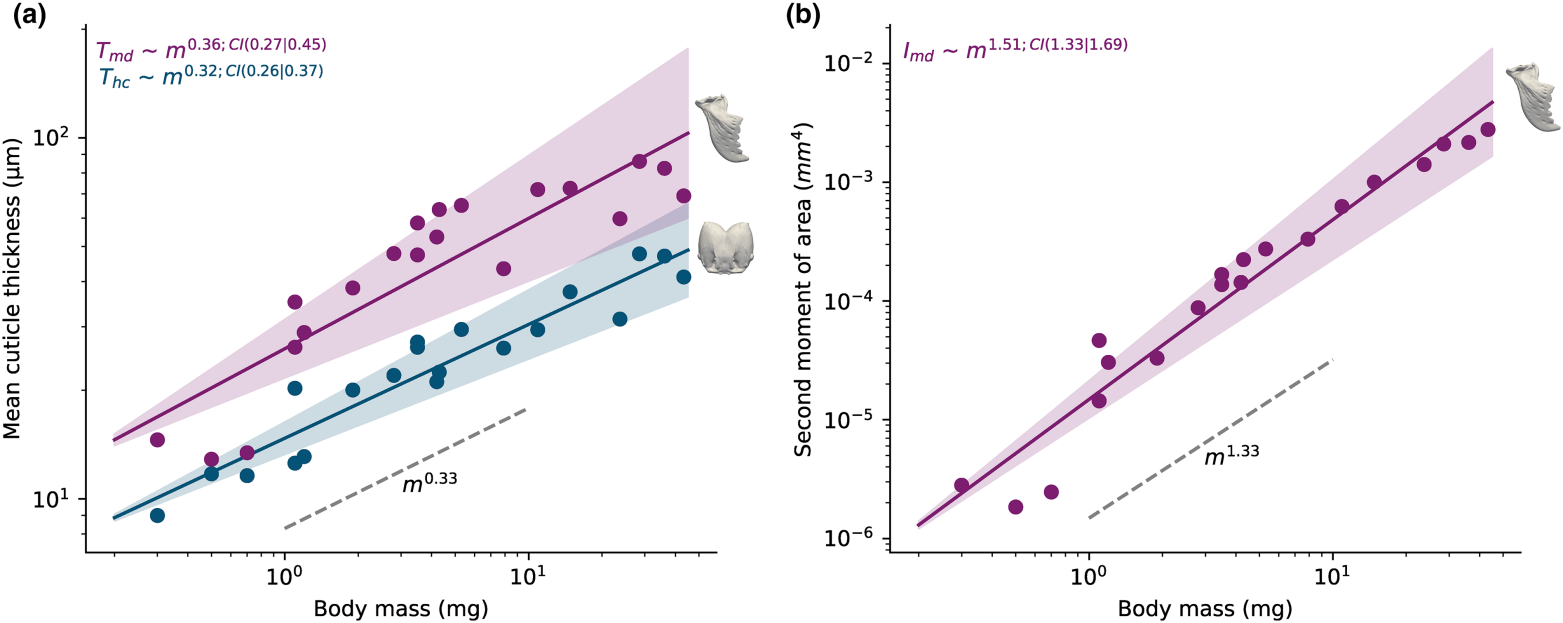
To assess internal shape changes, we extracted two morphological parameters of functional significance during biting from micro‐CT scans across the worker size range. (a) Mean cuticle thickness, *T*_md_ and *T*_hc_, of head capsules and mandibles, respectively, was consistent with isometry, which predicts *T*_hc_ *~ T*_md_ ~ *m* ^1/3^. (b) The allometry of the mandible second moment of area, *I*_md_, about the axis most functionally relevant during biting (see text) was consistent with isometry, which predicts *I*_md_ ~ *m* ^4/3^. The black lines represent the result of an ordinary least squares regression on log_10_‐transformed data; shading indicates the 95% confidence interval for the slope. Grey dashed lines indicate the null hypothesis of isometry.

The variation of head capsule and mandible thickness with media body mass did not differ significantly from isometry (head capsules: *b* = 0.32, 95% CI: 0.26–0.27; *R* ^2^ = 0.90; mandibles: *b* = 0.36, 95% CI: 0.26–0.45; *R* ^2^ = 0.79; Figure 4a). The mean second moment of area of the mandibles increased with *b* = 1.51, also in agreement with isometry (95% CI: 1.33–1.69; *R* ^2^ = 0.97; Figure 4b).

## 4 DISCUSSION

Colonies of eusocial insects often comprise multiple sterile phenotypes (Hölldobler & Wilson, 1990; Wills et al., 2018; Wilson, 1953, 1985). Such phenotypic plasticity may be achieved by variation of two critical parameters during ontogeny: the critical size at which growth stops; or the growth rates of body parts up to this point (Molet et al., 2014; Wheeler, 1991). Variation in critical size produces isometric phenotypes of variable size but identical shape. Variation in growth rates results in shape‐variation irrespective of size, and thus positive or negative allometry. In *Atta* leaf‐cutter ants, phenotypic plasticity has been taken to an extreme (e.g. Wetterer, 1999): sterile females may exist on a more or less continuous size‐spectrum, often spanning more than two orders of magnitude in body mass. Through quantitative 3D shape analysis, we demonstrated that sterile females in *A. vollenweideri* differ not only in size but also in shape: both mandibles and head capsules of minims appear morphologically distinct compared to those of medias; and mandibles and head capsules of sterile workers appear morphologically distinct from the reproductively active queen.

In support of this broad categorisation into three representative head capsule and mandible shape complexes, three lines of evidence may be presented. First, queen and minim head capsules and mandibles have a substantially larger total distance from the mean template shape than medias (Figures 2b and 3b). Second, head capsules and mandibles of minims, medias, and the queen occupy separate regions in the space span by the first three principal components (Figures 2c and 3c). Third, the difference in shape is evident to the eye unaided by statistical shape analysis (Figure 1). Our results thus align with earlier reports on mandible and head capsule 2D shape variation in workers of *A. colombica* Guerin‐Méneville, 1844, *A. bisphaerica* Forel, 1908, and *A. sexdens* (Linnaeus, 1758) (Franks & Norris, 1987; Hernandez & Caetano, 1995; Silva et al., 2016). But they contrast with conclusions based on a qualitative shape analysis in *A. laevigata* (Smith, 1858), which posited that worker mandibles differ solely in size (Hernandez & Caetano, 1995), and with the suggestion that *Atta* in general possess only a single worker caste which may be split into minors, medias and majors along arbitrary weight cut‐offs (e.g. Baroni Urbani, 1998).

### 4.1 Morphological and functional specialisation: distinct head and mandible morphs in *A. vollenweideri*

A main theme in the behavioural ecology of eusocial insects is the notion of division of labour: some tasks are exclusively or preferentially performed by specific individuals. These individuals are then often grouped into “castes”, in order to aid and guide further theoretical and empirical work (Fjerdingstad & Crozier, 2006; Wills et al., 2018), such as the study of colony ergonomics with “optimisation” theories borrowed from economics (Oster & Wilson, 1978; Wilson, 1968, 1979, 1985). Caste is used as a demarcation criterion in at least three different ways (Buschinger & Crozier, 1987; Silva & Feitosa, 2019; Villet, 1992; Wilson, 1953). Individuals of the same caste are thought to (i) have the same morphotype (a structural caste concept; e.g. Peeters & Crozier, 1988); (ii) perform the same task for extended periods of time (a functional caste concept; e.g. Michener, 1974; Buschinger & Crozier, 1987; Buschinger, 1990); or (iii) share both morphotype and task‐preference (a structural–functional caste concept; e.g. Wilson, 1968, 1990). Inspection of the statistical shape atlas suggested the existence of three structural head capsule and mandible morphs in female *A. vollenweideri*: (i) minims, (ii) medias, and (iii) the queen. Males were not investigated as part of this work, but may well represent an additional morph. The three shape categories seemingly coincide with functional specialisation: the queen is the only reproductively active female; minims are rarely found outside the nest, but are by far the dominant size‐class inside the fungal gardens (Figure S3; Muratore et al., 2023; Wetterer, 1999; Wilson, 1980); medias of all sizes actively partake in foraging (Clark, 2006; Muratore et al., 2023; Rudolph & Loudon, 1986; note that task preference can further vary with size within the medias, e.g. Camargo et al., 2007; Püffel, Meyer, et al., 2023). Notably, we did not find strong evidence for a separate soldier morph in *A. vollenweideri*, and indeed have observed that even the largest workers sometimes partake in foraging (see Wetterer, 1995 for similar results on *A. colombica*).

Two further subtle results are of note. First, workers with a body mass between 1‐2 mg occupy an intermediate region in shape space (Figures 2 and 3). Their mandibles have a shape that appears more similar to those of minims, but a head capsule shape more similar to that of medias (Figures 2b and 3b). Second, although all media head capsules and mandibles share a similarly small TdtMS, a systematic variation of shape with size persists (Figures 2 and 3). Thus, neither head capsules nor mandibles of medias of different sizes are strictly isometric variants of one another (see also Püffel et al., 2021). We submit two arguments why medias may nevertheless be considered a single distinct morph in head capsule and mandible shape: first, shape variation within this size group is qualitatively different to the variation across medias, minims and the queen, as it occurs dominantly along a single principal component (PC 1 for head capsules and PC 2 for mandibles; Figures 2c and 3c). Indeed, linear measurements of head capsule or mandible morphology, such as head width or blade length, can be related to size with a single allometric exponent (“monophasic allometry” sensu Wilson, 1953, see Figure 4 and Püffel et al., 2021), suggesting a common regulation of growth rates. Second, we observed workers across the size range from 2 to 45 mg actively engaging in foraging tasks, indicating no obvious material differences in task specialisation. In accordance with our laboratory observations, active foragers in the field ranged from 2 to 30 mg, with the majority of foragers falling between 5 and 15 mg (Röschard & Roces, 2002, 2003). Although larger workers cut tougher leaves (Clark, 2006; Evison & Ratnieks, 2007; Nichols‐Orians, 1991), this likely merely reflects a difference in ability – related to the absolutely larger bite forces they produce (see below) – and not a categorical functional specialisation.

Foraging is a broad role category which involves multiple elements, such as scouting, cutting, and leaf transport. Medias may thus have to be foraging generalists, and this requirement may constrain morphological differentiation. For example, young *Atta* foragers may initially cut plant tissue, but switch to leaf‐transport as their mandibles wear (Schofield et al., 2011). Morphological specialisation, in contrast, can permanently constrain behaviour (Noirot & Pasteels, 1987; Powell & Franks, 2006; Wilson, 1984). For example, the large mandibles of *Eciton* soldiers prevent them from carrying prey or brood, and they must be fed by other workers (Powell & Franks, 2006). The “elasticity” provided by purely functional specialisation avoids such permanent limitations, and enables colonies to respond quickly to environmental variation (Wheeler, 1986; Wilson, 1984). Retaining some independence between morphological and functional specialisation may therefore confer evolutionary advantages (Calabi, 1988). Some authors have argued that a morphological continuum within workers may be delineated in terms of sub‐castes (Baroni Urbani, 1998; Mirenda & Vinson, 1981; Moffett & Tobin, 1991), and the use of minims, medias, and majors based on arbitrarily defined size bins is common. However, the heuristic value of these categories, aside from a rough indication of body size, is not obvious.

### 4.2 Media mandible and head shape reflect mechanical demands of leaf‐cutting

The heads of minims and medias are distinguished primarily by the relative size of the occipital lobes. These lobes expand outwards and appear more curved in foragers, so leading to an increase in relative head volume, used to house a disproportionally larger mandible closer muscle (Figure 2a and Püffel et al., 2021). The change in head capsule shape arises partially from a faster growth of head width compared to head length (Figure 2a and Püffel et al., 2021), and this differential growth likely carries functional significance: it enables a disproportionate increase in physiological cross‐sectional area of the muscle, and thus a substantial increase in size‐specific bite force (Püffel et al., 2021). As a result, the largest foragers generate bite forces more than two times larger than the isometric expectation; this boost provides colonies with access a larger range of plant material, without requiring an extreme investment in even larger workers (Püffel, Roces, et al., 2023). The functional morphology of minim heads is considerably harder to assess, as the demands placed on head capsules by gardening or brood care are not well known. The relatively smaller heads of minims may be better suited for navigation in the tight fungal matrix, and the relaxed requirement to house large muscle to cut leaves may have permitted a decrease in relative head size. Notably, a size‐correlated difference between barrel‐ vs. heart‐shaped head capsules is a common feature not only in *Atta* (e.g. *A. sexdens*, see Wilson, 1980) but also in *Camponotus* (Galbán et al., 2021), *Pheidole* (Wetterer, 2012), and *Solenopsis* (Tschinkel, 2013), suggesting that some of the functional consequences may be shared across ant genera.

The mandibles of minims and medias are primarily distinguished by a variation in blade curvature, an elongation and reorientation of the apical incisor, and a thickening of the basal stem at the expense of the basal margin. Importantly, these differences are concentrated in mandible sections not impacted by wear, so making it unlikely that they merely reflect age differences (see Figure 3a; Püffel, Walthaus, et al., 2023; Schofield et al., 2011). Instead, each of the identified shape differences may be related to the mechanical demands imposed by leaf‐cutting. First, the change in orientation and elongation of the apical incisor likely enables the leading mandible to pierce the leaf lamina during cutting, so providing an anchor for the dragging mandible that performs the cut (Garrett et al., 2016; Schofield et al., 2011; Tautz et al., 1995). The elementary functional issue is that mandibles rotate approximately about one single axis (Kang et al., 2023), so that an inward orientation of a large tooth may be required to effectively exert the forces required for piercing during mandible closure. Second, the increased curvature of the mandible results in a superior mechanical performance in relevant loading scenarios: the stress induced by a combination of compressive and lateral tip forces is distributed more continuously along the length of the mandible, and the effective mandible stiffness is relatively insensitive to the orientation of the applied force (Bar‐On, 2019, 2023). Thus, curved mandibles are suitable for piercing and cutting irregularly shaped targets such as fruit or other animals during colony defence (Evison & Ratnieks, 2007; Helanterä & Ratnieks, 2008).

The concave depression of the masticatory margin, in turn, may represent an adaptation for the dragging mandible: the force transmitted by the mandible to the leaf lamina needs to be closely aligned with the lamina plane; even small deviations can lead to leaf bending or buckling. Keeping this force orientation constant throughout mandible closure is impossible with a straight masticatory margin, as the mandible rotates about an approximately fixed axis, but at least in theory possible with a curved masticatory margin. Third, the increased bulkiness of the basal stem likely reflects a response to the increase in the relative joint reaction forces (see also Klunk et al., 2021), arising from the disproportionate increase in muscle volume. In this context, it is noteworthy that neither the average thickness of the mandible cuticle and the head capsule nor the second moment of area grow with positive allometry (although, the latter almost does, see Figure 4). Perhaps the increased mechanical demand is met with changes in shape alone, but detailed computational analysis of the stress distribution across mandibles and head capsules of different shape is required to test this hypothesis (see e.g. Blanke, Schmitz, et al., 2017; Blanke, Watson, et al., 2017; Goyens et al., 2014, 2015; Hörnschemeyer et al., 2013; Klunk et al., 2021; Larabee et al., 2018).

### 4.3 The evolution of distinct worker morphs in *A. vollenweideri*

Many ant species show variation in worker size, and the degree of worker polymorphism generally correlates with both colony size and queen‐worker dimorphism, which are both greatest in highly eusocial ant species. Recent theoretical frameworks for caste development suggest caste differentiation is predominantly a question of size‐differences (Trible & Kronauer, 2017). In support of this conjecture, allometric growth rules are strongly conserved in both *Solenopsis* (Tschinkel, 2013) and *Pheidole* (Pie & Traniello, 2007), so that different shape almost always implies different size (see also Casadei‐Ferreira et al., 2022; Franks & Norris, 1987; Wills et al., 2018). Worker size is then expected to correlate with structural “queen‐likeness” (Trible & Kronauer, 2017) and indeed *Pheidole* soldiers even possess rudimental wing imaginal discs (Rajakumar et al., 2018). The smallest workers, in turn, should then represent the “pure” worker phenotype (Trible & Kronauer, 2017).

At least for mandibles and head capsules, this pattern is not confirmed in *A. vollenweideri*; both appear to move further away from the queen‐shape as workers increase in size. However, workers with a body mass between 1 and 2 mg may represent an intermediate state which combines minim‐ and media‐like morphological characteristics, or even a unique group with its own developmental program (Figures 2 and 3). The advantages of such a worker group in a leaf‐cutter colony may be task flexibility; intermediate workers may be able to process leaves but also perform more delicate tasks such as brood care efficiently. The existence of combined traits implies at least a weak decoupling of mandible and head shape during ontogeny. Indeed, we only found an intermediate correlation between the second principal component for mandible and head shape, reminiscent of results in *Pheidole*, a genus with a primarily dimorphic worker caste: *Pheidole* heads and mandibles are highly integrated but modular, so allowing the decoupled shape variation required for the evolution of distinct worker castes (Casadei‐Ferreira et al., 2021). A similar decoupling may have been crucial for the evolution of minims and medias in *A. vollenweideri*.

The evolution of novel worker morphs requires not only modularity and plasticity, but also a sufficiently strong evolutionary advantage (Friedman et al., 2020; Londe et al., 2015; Molet et al., 2012). A key advantage of very small workers (head width below 1 mm) is that they are cheap to produce; their evolution thus likely revolutionised the colony economy (Peeters & Ito, 2015). A unit volume of fungus typically requires a minimum biomass of ants to ensure stability (Farias et al., 2020; Weber, 1972). Perhaps minims are an adaptation to cheaply and rapidly produce fungal maintenance workers, and thus a continuation of the general evolutionary trend of worker miniaturisation (Peeters & Ito, 2015). Consistent with this idea, minim workers have been shown to be more successful at maintaining a fungal garden (Abramowski et al., 2011), perhaps facilitated by their distinct head and mandible morphology; and indeed, body size influences task preference when it comes to fungal garden maintenance: smaller ants primarily groom and remove small pieces, whereas large ants primarily weed and remove large pieces of fungus, suggesting that mandible morphology may influence fungal maintenance performance more broadly (Abramowski et al., 2011; Currie & Stuart, 2001). Further work is required to determine whether minims, medias, or the intermediate phenotype represent the ancestral worker morphology in *A. vollenweideri* and whether other species in the genus have a similar or different number of morphologically distinct worker morphs.

## 5 OUTLOOK

We have used large diffeomorphic metric mapping to study 3D shape variation of head capsules and mandibles in *A. vollenweideri* leaf‐cutter ants. The key advantage of this technique over landmark‐based morphometry is that it in principle compares shape without requiring substantial user input. It thus becomes possible to characterise minute variations in 3D shape with high resolution and in minimal time. A limitation is that surface deformation approaches such as LDDMM may not be appropriate when morphology varies greatly, such as between triangular ant mandibles and elongated trap‐jaw mandibles, and may thus be limited to studies with closely related species. Further work is needed to assess how well the approach works across highly distinct morphologies. A particularly attractive feature of a shape atlas is that it enables the generation of detailed 3D shape averages. Expanding the shape atlas to include other body parts of *A. vollenweideri* workers, such as the legs or the thorax, is necessary to achieve a complete understanding of the co‐variation between size and shape in *Atta*. Similarly, including other *Atta* species, or conducting broad comparative studies across disparate clades, presents the tantalising opportunity to estimate characteristic mean shapes for different groups, which may then be used, for example, in finite element simulations to investigate the functional significance of different stereotypic forms in more detail. Such quantitative approaches that tie in complex 3D shape variation with mechanical performance will help to identify evolutionary drivers and constraints of worker polymorphism in ants, and elucidate the functional significance of shape variation across the insect tree of life in general.

## AUTHOR CONTRIBUTIONS

**Natalie Imirzian:** Conceptualization (equal); formal analysis (lead); investigation (equal); methodology (lead); writing – original draft (lead); writing – review and editing (equal). **Frederik Püffel:** Formal analysis (supporting); investigation (equal); methodology (supporting); writing – review and editing (equal). **Flavio Roces:** Methodology (supporting); resources (lead); writing – review and editing (equal). **David Labonte:** Conceptualization (equal); funding acquisition (lead); supervision (lead); writing – original draft (supporting); writing – review and editing (equal).

## CONFLICT OF INTEREST STATEMENT

The authors declare no competing interests.

## Supporting information


Appendix S1

## Data Availability

The data that support the findings of this study are openly available at figshare with the link https://doi.org/10.6084/m9.figshare.22795841. Mesh files from the study are available at https://skfb.ly/oFPAM. Code to run the 3D analysis is found at https://github.com/nimirz/ShapeAtlas.

## References

[ece311236-bib-0001] Abramowski, D. , Currie, C. R. , & Poulsen, M. (2011). Caste specialization in behavioral defenses against fungus garden parasites in *Acromyrmex octospinosus* leaf‐cutting ants. Insectes Sociaux, 58(1), 65–75. 10.1007/s00040-010-0117-y

[ece311236-bib-0002] Adams, D. , Rohlf, F. J. , & Slice, D. (2013). A field comes of age: Geometric morphometrics in the 21st century. Hystrix, the Italian Journal of Mammalogy, 24(1), 7–14.

[ece311236-bib-0144] Ahrens, J. , Geveci, B. , & Law, C. (2005). ParaView: An end‐user tool for large data visualization. Visualization handbook. Elsevier.

[ece311236-bib-0003] Aibekova, L. , Keller, R. A. , Katzke, J. , Allman, D. M. , Hita‐Garcia, F. , Labonte, D. , Narendra, A. , & Economo, E. P. (2023). Parallel and divergent morphological adaptations underlying the evolution of jumping ability in ants. Integrative Organismal Biology, 5(1), obad026. 10.1093/iob/obad026 37545740 PMC10401624

[ece311236-bib-0004] Aneja, D. , Vora, S. R. , Camci, E. D. , Shapiro, L. G. , & Cox, T. C. (2015). Automated detection of 3D landmarks for the elimination of non‐biological variation in geometric morphometric analyses. In 2015 IEEE 28th International symposium on computer‐based medical systems (pp. 78–83). IEEE. 10.1109/CBMS.2015.86 PMC4526271 26258171

[ece311236-bib-0005] Bardua, C. , Felice, R. N. , Watanabe, A. , Fabre, A. C. , & Goswami, A. (2019). A practical guide to sliding and surface Semilandmarks in morphometric analyses. Integrative Organismal Biology, 1(1), obz016. 10.1093/iob/obz016 33791531 PMC7780474

[ece311236-bib-0006] Bar‐On, B. (2019). On the form and bio‐mechanics of venom‐injection elements. Acta Biomaterialia, 85, 263–271. 10.1016/j.actbio.2018.12.030 30583109

[ece311236-bib-0007] Bar‐On, B. (2023). The effect of structural curvature on the load‐bearing characteristics of biomechanical elements. Journal of the Mechanical Behavior of Biomedical Materials, 138, 105569. 10.1016/j.jmbbm.2022.105569 36549249

[ece311236-bib-0008] Baroni Urbani, C. (1998). The number of castes in ants, where major is smaller than minor and queens wear the shield of the soldiers. Insectes Sociaux, 45(3), 315–333. 10.1007/s000400050091

[ece311236-bib-0009] Bernadou, A. , Kramer, B. H. , & Korb, J. (2021). Major evolutionary transitions in social insects, the importance of worker sterility and life history trade‐offs. Frontiers in Ecology and Evolution, 9, 732907. 10.3389/fevo.2021.732907

[ece311236-bib-0010] Beshers, S. N. , & Fewell, J. H. (2001). Models of division of labor in social insects. Annual Review of Entomology, 46, 413–440. 10.1146/annurev.ento.46.1.413 11112175

[ece311236-bib-0011] Blanke, A. , Schmitz, H. , Patera, A. , Dutel, H. , & Fagan, M. J. (2017). Form–function relationships in dragonfly mandibles under an evolutionary perspective. Journal of the Royal Society Interface, 14(128), 20161038. 10.1098/rsif.2016.1038 28330989 PMC5378138

[ece311236-bib-0012] Blanke, A. , Watson, P. J. , Holbrey, R. , & Fagan, M. J. (2017). Computational biomechanics changes our view on insect head evolution. Proceedings of the Royal Society B: Biological Sciences, 284(1848), 20162412. 10.1098/rspb.2016.2412 PMC5310608 28179518

[ece311236-bib-0013] Boehm, B. , Rautschka, M. , Quintana, L. , Raspopovic, J. , Jan, Ž. , & Sharpe, J. (2011). A landmark‐free morphometric staging system for the mouse limb bud. Development, 138(6), 1227–1234. 21307091 10.1242/dev.057547 PMC3042875

[ece311236-bib-0014] Bône, A. , Louis, M. , Martin, B. , & Durrleman, S. (2018). Deformetrica 4: An open‐source software for statistical shape analysis. In M. Reuter , H. Lombaert , B. Paniagua , M. Lüthi , & B. Egger (Eds.), Shape in medical imaging. Lecture notes in computer science (pp. 3–13). Springer International Publishing. 10.1007/978-3-030-04747-4_1

[ece311236-bib-0015] Booher, D. B. , Gibson, J. C. , Liu, C. , Longino, J. T. , Fisher, B. L. , Janda, M. , Narula, N. , Toulkeridou, E. , Mikheyev, A. S. , Suarez, A. V. , & Economo, E. P. (2021). Functional innovation promotes diversification of form in the evolution of an ultrafast trap‐jaw mechanism in ants. PLoS Biology, 19(3), e3001031. 10.1371/journal.pbio.3001031 33651798 PMC7924744

[ece311236-bib-0016] Bookstein, F. L. (1991). Morphometric tools for landmark data: Geometry and biology. Cambridge University Press.

[ece311236-bib-0145] Boudinot, B. E. , Moosdorf, O. T. D. , Beutel, R. G. , & Richter, A. (2021). Anatomy and evolution of the head of *Dorylus helvolus* (Formicidae: Dorylinae): Patterns of sex‐ and caste‐limited traits in the sausagefly and the driver ant. Journal of Morphology, 282(11), 1616–1658. 10.1002/jmor.21410 34427942

[ece311236-bib-0017] Boyer, D. M. , Lipman, Y. , St. Clair, E. , Puente, J. , Patel, B. A. , Funkhouser, T. , Jernvall, J. , & Daubechies, I. (2011). Algorithms to automatically quantify the geometric similarity of anatomical surfaces. Proceedings of the National Academy of Sciences of the United States of America, 108(45), 18221–18226. 10.1073/pnas.1112822108 22025685 PMC3215009

[ece311236-bib-0018] Braga, J. , Zimmer, V. , Dumoncel, J. , Samir, C. , de Beer, F. , Zanolli, C. , Pinto, D. , Rohlf, F. J. , & Grine, F. E. (2019). Efficacy of diffeomorphic surface matching and 3D geometric morphometrics for taxonomic discrimination of Early Pleistocene hominin mandibular molars. Journal of Human Evolution, 130, 21–35. 10.1016/j.jhevol.2019.01.009 31010541

[ece311236-bib-0019] Buschinger, A. (1990). Regulation of worker and queen formation in ants with special reference to reproduction and colony development. In W. Engels (Ed.), Social insects: An evolutionary approach to castes and reproduction (pp. 37–57). Springer. 10.1007/978-3-642-74490-7_4

[ece311236-bib-0020] Buschinger, A. , & Crozier, R. (1987). Towards a unified reproductive biology of the Hymenoptera, Chemistry and biology of social insects. Verlag J. Peperny. p. 251.

[ece311236-bib-0021] Calabi, P. (1988). Behavioral flexibility in hymenoptera: A re‐examination of the concept of caste. In J. C. Trager (Ed.), Advances in Myrmecology (pp. 237–258). Brill.

[ece311236-bib-0022] Camargo, R. S. , Forti, L. C. , Lopes, J. F. S. , Andrade, A. P. P. , & Ottati, A. L. T. (2007). Age polyethism in the leaf‐cutting ant Acromyrmex subterraneus brunneus Forel, 1911 (Hym., Formicidae). Journal of Applied Entomology, 131(2), 139–145. 10.1111/j.1439-0418.2006.01129.x

[ece311236-bib-0023] Casadei‐Ferreira, A. , Feitosa, R. M. , & Pie, M. R. (2022). Size and shape in the evolution of the worker head in Pheidole ants (Hymenoptera: Formicidae). Journal of Zoology, 317(4), 270–282. 10.1111/jzo.12978

[ece311236-bib-0024] Casadei‐Ferreira, A. , Friedman, N. R. , Economo, E. P. , Pie, M. R. , & Feitosa, R. M. (2021). Head and mandible shapes are highly integrated yet represent two distinct modules within and among worker subcastes of the ant genus Pheidole. Ecology and Evolution, 11(11), 6104–6118. 10.1002/ece3.7422 34141206 PMC8207162

[ece311236-bib-0025] Cherrett, J. M. (1972). Some factors involved in the selection of vegetable substrate by Atta cephalotes (L.) (Hymenoptera: Formicidae) in tropical rain Forest. Journal of Animal Ecology, 41(3), 647–660. 10.2307/3200

[ece311236-bib-0026] Cignoni, P. , Callieri, M. , Corsini, M. , Dellepiane, M. , Ganovelli, F. , & Ranzuglia, G. (2008). MeshLab: An open‐source mesh processing tool. In V. Scarano , R. D. Chiara , & U. Erra (Eds.), Eurographics Italian chapter conference. The Eurographics Association.

[ece311236-bib-0027] Clark, E. (2006). Dynamic matching of forager size to resources in the continuously polymorphic leaf‐cutter ant, *Atta colombica* (Hymenoptera, Formicidae). Ecological Entomology, 31(6), 629–635. 10.1111/j.1365-2311.2006.00826.x

[ece311236-bib-0028] Cole, B. J. (2020). Comparative advantage and caste evolution. Evolution, 74(3), 655–659. 10.1111/evo.13928 31953841

[ece311236-bib-0029] Corn, M. L. (1980). Polymorphism and polyethism in the neotropical ant *Cephalotes atratus* (L.). Insectes Sociaux, 27(1), 29–42. 10.1007/BF02224519

[ece311236-bib-0030] Currie, C. R. , & Stuart, A. E. (2001). Weeding and grooming of pathogens in agriculture by ants. Proceedings of the Royal Society of London. Series B: Biological Sciences, 268(1471), 1033–1039. 10.1098/rspb.2001.1605 PMC1088705 11375087

[ece311236-bib-0031] Doube, M. , Kłosowski, M. M. , Arganda‐Carreras, I. , Cordelières, F. P. , Dougherty, R. P. , Jackson, J. S. , Schmid, B. , Hutchinson, J. R. , & Shefelbine, S. J. (2010). BoneJ: Free and extensible bone image analysis in ImageJ. Bone, 47(6), 1076–1079. 10.1016/j.bone.2010.08.023 20817052 PMC3193171

[ece311236-bib-0033] Durrleman, S. , Prastawa, M. , Charon, N. , Korenberg, J. R. , Joshi, S. , Gerig, G. , & Trouvé, A. (2014). Morphometry of anatomical shape complexes with dense deformations and sparse parameters. NeuroImage, 101, 35–49. 10.1016/j.neuroimage.2014.06.043 24973601 PMC4871626

[ece311236-bib-0143] Durrleman, S. , Prastawa, M. , Korenberg, J. R. , Joshi, S. , Trouvé, A. , & Gerig, G. (2012). Topology preserving atlas construction from shape data without correspondence using sparse parameters. Lecture Notes in Computer Science, 15(Pt 3), 223–230. 10.1007/978-3-642-33454-2_28 PMC3758250 23286134

[ece311236-bib-0034] Evison, S. E. F. , & Ratnieks, F. L. W. (2007). New role for majors in Atta leafcutter ants. Ecological Entomology, 32(5), 451–454. 10.1111/j.1365-2311.2007.00877.x

[ece311236-bib-0035] Farias, A. P. , Camargo, R. S. , Andrade Sousa, K. K. , Caldato, N. , & Forti, L. C. (2020). Nest architecture and Colony growth of *Atta bisphaerica* grass‐cutting ants. Insects, 11(11), 741. 10.3390/insects11110741 33137875 PMC7693648

[ece311236-bib-0036] Feener, D. H. , Lighton, J. R. B. , & Bartholomew, G. A. (1988). Curvilinear Allometry, energetics and foraging ecology: A comparison of leaf‐cutting ants and Army ants. Functional Ecology, 2(4), 509–520. 10.2307/2389394

[ece311236-bib-0037] Fjerdingstad, E. J. , & Crozier, R. H. (2006). The evolution of worker caste diversity in social insects. The American Naturalist, 167(3), 390–400. 10.1086/499545 16673347

[ece311236-bib-0038] Franks, N. R. (1985). Reproduction, foraging efficiency and worker polymorphism in army ants. In Experimental behavioural ecology and sociobiology. (Fortschritte der Zoologie) (pp. 91–107). Gustav Fischer Verlag.

[ece311236-bib-0039] Franks, N. R. , & Norris, P. J. (1987). Constraints on the division of labour in ants: D'Arcy Thompson's cartesian transformations applied to worker polymorphism. In J. M. Pasteels & J. L. Deneubourg (Eds.), From individual to collective behavior in social insects. Les Treilles Workshop. Birkhauser. https://scholar.google.com/scholar_lookup?title=Constraints+on+the+division+of+labour+in+ants%3A+D%27Arcy+Thompson%27s+cartesian+transformations+applied+to+worker+polymorphism&author=Franks%2C+N.R.&publication_year=1987

[ece311236-bib-0040] Friedman, N. R. , Lecroq Bennet, B. , Fischer, G. , Sarnat, E. M. , Huang, J. P. , Knowles, L. L. K. , & Economo, E. P. (2020). Macroevolutionary integration of phenotypes within and across ant worker castes. Ecology and Evolution, 10(17), 9371–9383. 10.1002/ece3.6623 32953067 PMC7487254

[ece311236-bib-0041] Galbán, A. , Cuezzo, F. , & Torréns, J. (2021). The pronotum of worker of *Camponotus borellii* Emery (hymenoptera: Formicidae): How can it affect performance of the head, work division, and development of the worker caste? Neotropical Entomology, 50(1), 78–89. 10.1007/s13744-020-00828-0 33501632

[ece311236-bib-0042] Garrett, R. W. , Carlson, K. A. , Goggans, M. S. , Nesson, M. H. , Shepard, C. A. , & Schofield, R. M. (2016). Leaf processing behaviour in Atta leafcutter ants: 90% of leaf cutting takes place inside the nest, and ants select pieces that require less cutting. Royal Society Open Science, 3(1), 150111. 10.1098/rsos.150111 26909161 PMC4736916

[ece311236-bib-0043] Goswami, A. , Watanabe, A. , Felice, R. N. , Bardua, C. , Fabre, A. C. , & Polly, P. D. (2019). High‐density morphometric analysis of shape and integration: The good, the bad, and the not‐really‐a‐problem. Integrative and Comparative Biology, 59(3), 669–683. 10.1093/icb/icz120 31243431 PMC6754122

[ece311236-bib-0044] Gould, S. J. (1966). Allometry and size in ontogeny and phylogeny. Biological Reviews, 41(4), 587–638. 10.1111/j.1469-185X.1966.tb01624.x 5342162

[ece311236-bib-0045] Goyens, J. , Dirckx, J. , & Aerts, P. (2015). Built to fight: Variable loading conditions and stress distribution in stag beetle jaws. Bioinspiration & Biomimetics, 10(4), 46006. 10.1088/1748-3190/10/4/046006 26133578

[ece311236-bib-0046] Goyens, J. , Soons, J. , Aerts, P. , & Dirckx, J. (2014). Finite‐element modelling reveals force modulation of jaw adductors in stag beetles. Journal of the Royal Society Interface, 11(101), 20140908. 10.1098/rsif.2014.0908 25297317 PMC4223912

[ece311236-bib-0047] Helanterä, H. , & Ratnieks, F. L. W. (2008). Geometry explains the benefits of division of labour in a leafcutter ant. Proceedings of the Royal Society B: Biological Sciences, 275(1640), 1255–1260. 10.1098/rspb.2008.0024 PMC2602677 18319212

[ece311236-bib-0048] Hernandez, J. V. , & Caetano, F. H. (1995). Charaterization of the mandible and mandibular glands in different castes of the leaf‐cutting ant *Atta laevigata* (F. Smith) (Hymenoptera: Formicidae)using scanning electron microscopy. Boletín de Entomología Venezolana, 10, 51–56.

[ece311236-bib-0049] Hita‐Garcia, F. , Lieberman, Z. , Audisio, T. L. , Liu, C. , & Economo, E. P. (2019). Revision of the highly specialized ant genus Discothyrea (Hymenoptera: Formicidae) in the Afrotropics with X‐ray microtomography and 3D Cybertaxonomy. Insect Systematics and Diversity, 3(6), 5. 10.1093/isd/ixz015

[ece311236-bib-0050] Hölldobler, B. , & Wilson, E. O. (1990). The ants. Harvard University Press.

[ece311236-bib-0051] Hörnschemeyer, T. , Bond, J. , & Young, P. G. (2013). Analysis of the functional morphology of mouthparts of the beetle *Priacma serrata*, and a discussion of possible food sources. Journal of Insect Science, 13(1), 126. 10.1673/031.013.12601 24786670 PMC4014034

[ece311236-bib-0052] Huxley, J. S. (1924). Constant differential growth‐ratios and their significance. Nature, 114(2877), 895–896. 10.1038/114895a0

[ece311236-bib-0053] Jasmin, J.‐N. , & Devaux, C. (2015). Laterality of leaf cutting in the attine ant Acromyrmexechinatior. Insectes Sociaux, 62(1), 109–114. 10.1007/s00040-014-0379-x

[ece311236-bib-0150] Kang, V. , Püffel, F. , & Labonte, D. (2023). Three‐dimensional kinematics of leaf‐cutter ant mandibles: Not all dicondylic joints are simple hinges. Philosophical Transactions of the Royal Society B, 378(1891), 20220546. 10.1098/rstb.2022.0546 PMC10577034 37839448

[ece311236-bib-0054] Keller, R. A. (2011). A phylogenetic analysis of ant morphology (Hymenoptera: Formicidae) with special reference to the Poneromorph subfamilies. Bulletin of the American Museum of Natural History, 2011(355), 1–90. 10.1206/355.1

[ece311236-bib-0055] Klingenberg, C. P. (2010). Evolution and development of shape: Integrating quantitative approaches. Nature Reviews Genetics, 11(9), 623–635. 10.1038/nrg2829 20697423

[ece311236-bib-0056] Klunk, C. L. , Argenta, M. A. , Casadei‐Ferreira, A. , Economo, E. P. , & Pie, M. R. (2021). Mandibular morphology, task specialization and bite mechanics in Pheidole ants (Hymenoptera: Formicidae). Journal of the Royal Society Interface, 18(179), 20210318. 10.1098/rsif.2021.0318 34102082 PMC8187013

[ece311236-bib-0057] Knapp, M. (2012). Preservative fluid and storage conditions alter body mass estimation in a terrestrial insect. Entomologia Experimentalis et Applicata, 143(2), 185–190. 10.1111/j.1570-7458.2012.01247.x

[ece311236-bib-0058] Koehl, P. , & Hass, J. (2015). Landmark‐free geometric methods in biological shape analysis. Journal of the Royal Society Interface, 12(113), 20150795. 10.1098/rsif.2015.0795 26631331 PMC4707851

[ece311236-bib-0059] Kronauer, D. J. (2020). Army ants: nature's ultimate social hunters. Harvard University Press.

[ece311236-bib-0060] Kundanati, L. , Chahare, N. R. , Jaddivada, S. , Karkisaval, A. G. , Sridhar, R. , Pugno, N. M. , & Gundiah, N. (2020). Cutting mechanics of wood by beetle larval mandibles. Journal of the Mechanical Behavior of Biomedical Materials, 112, 104027. 10.1016/j.jmbbm.2020.104027 32916590

[ece311236-bib-0061] Larabee, F. J. , Smith, A. A. , & Suarez, A. V. (2018). Snap‐jaw morphology is specialized for high‐speed power amplification in the Dracula ant, *Mystrium camillae* . Royal Society Open Science, 5(12), 181447. 10.1098/rsos.181447 30662749 PMC6304126

[ece311236-bib-0062] Londe, S. , Monnin, T. , Cornette, R. , Debat, V. , Fisher, B. L. , & Molet, M. (2015). Phenotypic plasticity and modularity allow for the production of novel mosaic phenotypes in ants. EvoDevo, 6(1), 36. 10.1186/s13227-015-0031-5 26629324 PMC4666092

[ece311236-bib-0063] Mayhe‐Nunes, A. J. , & Caetano, F. H. (1994). Ultramorphology of, and comparison between, the mandibular gland and mandibles of two species of Acromyrmex (Hymenoptera, Formicidae). Natura, 19, 17–27.

[ece311236-bib-0064] Meijering, E. H. , Niessen, W. J. , & Viergever, M. A. (2001). Quantitative evaluation of convolution‐based methods for medical image interpolation. Medical Image Analysis, 5(2), 111–126. 11516706 10.1016/s1361-8415(00)00040-2

[ece311236-bib-0065] Michener, C. D. (1974). The social behavior of the bees: A comparative study. Harvard University Press.

[ece311236-bib-0066] Mirenda, J. T. , & Vinson, S. B. (1981). Division of labour and specification of castes in the red imported fire ant Solenopsis invicta buren. Animal Behaviour, 29(2), 410–420. 10.1016/S0003-3472(81)80100-5

[ece311236-bib-0142] Mitteroecker, P. , Gunz, P. , Windhager, S. , & Schaefer, K. (2013). A brief review of shape, form, and allometry in geometric morphometrics, with applications to human facial morphology. Hystrix, the Italian Journal of Mammalogy, 24(1), 59–66.

[ece311236-bib-0067] Moffett, M. W. , & Tobin, J. E. (1991). Physical castes in ant workers: A problem for *Daceton armigerum* and other ants. Psyche, 98(4), 283–292.

[ece311236-bib-0068] Molet, M. , Maicher, V. , & Peeters, C. (2014). Bigger helpers in the ant *Cataglyphis bombycina*: Increased worker polymorphism or novel soldier caste? PLoS One, 9(1), e84929. 10.1371/journal.pone.0084929 24404196 PMC3880325

[ece311236-bib-0069] Molet, M. , Wheeler, D. E. , & Peeters, C. (2012). Evolution of novel mosaic castes in ants: Modularity, phenotypic plasticity, and colonial buffering. The American Naturalist, 180(3), 328–341. 10.1086/667368 22854076

[ece311236-bib-0070] Muratore, I. , Ilieş, I. , Huzar, A. K. , Zaidi, F. H. , & Traniello, J. F. A. (2023). Morphological evolution and the behavioral organization of agricultural division of labor in the leafcutter ant *Atta cephalotes* . Behavioral Ecology and Sociobiology, 77(6), 70.

[ece311236-bib-0071] Nichols‐Orians, C. M. (1991). Environmentally induced differences in plant traits: Consequences for susceptibility to a leaf‐cutter ant. Ecology, 72(5), 1609–1623. 10.2307/1940961

[ece311236-bib-0072] Noirot, C. , & Pasteels, J. M. (1987). Ontogenetic development and evolution of the worker caste in termites. Experientia, 43(8), 851–860. 10.1007/BF01951642

[ece311236-bib-0073] Oster, G. F. , & Wilson, E. O. (1978). Caste and ecology in the social insects. Princeton University Press. 740003

[ece311236-bib-0074] Pedregosa, F. , Varoquaux, G. , Gramfort, A. , Michel, V. , Thirion, B. , Grisel, O. , Blondel, M. , Prettenhofer, P. , Weiss, R. , Dubourg, V. , Vanderplas, J. , Passos, A. , Cournapeau, D. , Brucher, M. , Perrot, M. , & Duchesna, É. (2011). Scikit‐learn: Machine learning in python. Journal of Machine Learning Research, 12, 2825–2830.

[ece311236-bib-0075] Peeters, C. , & Crozier, R. H. (1988). Caste and reproduction in ants: Not all mated egg‐layers are “queens”. Psyche, 95(3–4), 283–288.

[ece311236-bib-0076] Peeters, C. , & Ito, F. (2015). Wingless and dwarf workers underlie the ecological success of ants (Hymenoptera: Formicidae). Myrmecological News, 21, 117–130. 10.5281/zenodo.845751

[ece311236-bib-0077] Peeters, C. , Keller, R. A. , Khalife, A. , Fischer, G. , Katzke, J. , Blanke, A. , & Economo, E. P. (2020). The loss of flight in ant workers enabled an evolutionary redesign of the thorax for ground labour. Frontiers in Zoology, 17(1), 33. 10.1186/s12983-020-00375-9 33088333 PMC7574298

[ece311236-bib-0078] Percival, C. J. , Devine, J. , Darwin, B. C. , Liu, W. , van Eede, M. , Henkelman, R. M. , & Hallgrimsson, B. (2019). The effect of automated landmark identification on morphometric analyses. Journal of Anatomy, 234(6), 917–935. 10.1111/joa.12973 30901082 PMC6539672

[ece311236-bib-0079] Percival, C. J. , Green, R. , Marcucio, R. , & Hallgrímsson, B. (2014). Surface landmark quantification of embryonic mouse craniofacial morphogenesis. BMC Developmental Biology, 14(1), 31. 10.1186/1471-213X-14-31 25059626 PMC4222779

[ece311236-bib-0080] Pie, M. R. , & Traniello, J. F. A. (2007). Morphological evolution in a hyperdiverse clade: The ant genus Pheidole. Journal of Zoology, 271(1), 99–109. 10.1111/j.1469-7998.2006.00239.x

[ece311236-bib-0081] Plum, F. , & Labonte, D. (2021). scAnt—An open‐source platform for the creation of 3D models of arthropods (and other small objects). PeerJ, 9, e11155. 10.7717/peerj.11155 33954036 PMC8048404

[ece311236-bib-0082] Pomidor, B. J. , Makedonska, J. , & Slice, D. E. (2016). A landmark‐free method for three‐dimensional shape analysis. PLoS One, 11(3), e0150368. 10.1371/journal.pone.0150368 26953573 PMC4783062

[ece311236-bib-0083] Powell, S. (2008). Ecological specialization and the evolution of a specialized caste in Cephalotes ants. Functional Ecology, 22(5), 902–911. 10.1111/j.1365-2435.2008.01436.x

[ece311236-bib-0084] Powell, S. , & Franks, N. R. (2006). Ecology and the evolution of worker morphological diversity: A comparative analysis with Eciton Army ants. Functional Ecology, 20(6), 1105–1114.

[ece311236-bib-0085] Powell, S. , Price, S. L. , & Kronauer, D. J. C. (2020). Trait evolution is reversible, repeatable, and decoupled in the soldier caste of turtle ants. Proceedings of the National Academy of Sciences of the United States of America, 117(12), 6608–6615. 10.1073/pnas.1913750117 32152103 PMC7104247

[ece311236-bib-0086] Püffel, F. , Meyer, L. , Imirzian, N. , Roces, F. , Johnston, R. , & Labonte, D. (2023). Developmental biomechanics and age polyethism in leaf‐cutter ants. Proceedings of the Royal Society B: Biological Sciences, 290(2000), 20230355. 10.1098/rspb.2023.0355 PMC10265030 37312549

[ece311236-bib-0087] Püffel, F. , Pouget, A. , Liu, X. , Zuber, M. , van de Kamp, T. , Roces, F. , & Labonte, D. (2021). Morphological determinants of bite force capacity in insects: A biomechanical analysis of polymorphic leaf‐cutter ants. Journal of the Royal Society Interface, 18(182), 20210424. 10.1098/rsif.2021.0424 34493090 PMC8424304

[ece311236-bib-0141] Püffel, F. , Roces, F. , & Labonte, D. (2023). Strong positive allometry of bite force in leaf‐cutter ants increases the range of cuttable plant tissues. Journal of Experimental Biology, 226(13), jeb245140. 10.1242/jeb.245140 37293932 PMC10357016

[ece311236-bib-0089] Püffel, F. , Walthaus, O. K. , Kang, V. , & Labonte, D. (2023). Biomechanics of cutting: Sharpness, wear sensitivity, and the scaling of cutting forces in leaf‐cutter ant mandibles. Philosophical Transactions of the Royal Society B: Biological Sciences, 378(1891), 20220547. 10.1098/rstb.2022.0547 PMC10577030 37839449

[ece311236-bib-0090] Rajakumar, R. , Koch, S. , Couture, M. , Favé, M. J. , Lillico‐Ouachour, A. , Chen, T. , de Blasis, G. , Rajakumar, A. , Ouellette, D. , & Abouheif, E. (2018). Social regulation of a rudimentary organ generates complex worker‐caste systems in ants. Nature, 562(7728), 574–577. 10.1038/s41586-018-0613-1 30305737

[ece311236-bib-0091] Richter, A. , Boudinot, B. , Yamamoto, S. , Katzke, J. , & Beutel, R. G. (2022). The first reconstruction of the head anatomy of a cretaceous insect, *Gerontoformica gracilis* (Hymenoptera: Formicidae), and the early evolution of ants. Insect Systematics and Diversity, 6(5), 4. 10.1093/isd/ixac013

[ece311236-bib-0092] Richter, A. , Garcia, F. H. , Keller, R. , Billen, J. , Economo, E. P. , & Beutel, R. (2020). Comparative analysis of worker head anatomy of Formica and Brachyponera (Hymenoptera: Formicidae). Arthropod Systematics & Phylogeny, 78, 133–170.

[ece311236-bib-0093] Richter, A. , Keller, R. A. , Rosumek, F. B. , Economo, E. P. , Hita Garcia, F. , & Beutel, R. G. (2019). The cephalic anatomy of workers of the ant species Wasmannia affinis (Formicidae, Hymenoptera, Insecta) and its evolutionary implications. Arthropod Structure & Development, 49, 26–49. 10.1016/j.asd.2019.02.002 30738181

[ece311236-bib-0094] Richter, A. , Garcia, F. H. , Keller, R. A. , Billen, J. , Katzke, J. , Boudinot, B. E. , Economo, E. P. , & Beutel, R. G. (2021). The head anatomy of *Protanilla lini* (Hymenoptera: Formicidae: Leptanillinae), with a hypothesis of their mandibular movement. Myrmecological News, 31, 85–114. 10.25849/myrmecol.news_031:085

[ece311236-bib-0095] Rohlf, F. J. (1990). Morphometrics. Annual Review of Ecology and Systematics, 21, 299–316.

[ece311236-bib-0096] Rohlf, F. J. , & Marcus, L. F. (1993). A revolution morphometrics. Trends in Ecology & Evolution, 8(4), 129–132. 10.1016/0169-5347(93)90024-J 21236128

[ece311236-bib-0097] Rohlf, F. J. , & Slice, D. (1990). Extensions of the Procrustes method for the optimal superimposition of landmarks. Systematic Biology, 39(1), 40–59. 10.2307/2992207

[ece311236-bib-0098] Röschard, J. , & Roces, F. (2002). The effect of load length, width and mass on transport rate in the grass‐cutting ant *Atta vollenweideri* . Oecologia, 131(2), 319–324. 10.1007/s00442-002-0882-z 28547700

[ece311236-bib-0099] Röschard, J. , & Roces, F. (2003). Fragment‐size determination and size‐matching in the grass‐cutting ant Atta vollenweideri depend on the distance from the nest. Journal of Tropical Ecology, 19(6), 647–653. 10.1017/S0266467403006047

[ece311236-bib-0100] Rudolph, S. G. , & Loudon, C. (1986). Load size selection by foraging leaf‐cutter ants (*Atta cephalotes*). Ecological Entomology, 11(4), 401–410. 10.1111/j.1365-2311.1986.tb00319.x

[ece311236-bib-0101] Schindelin, J. , Arganda‐Carreras, I. , Frise, E. , Kaynig, V. , Longair, M. , Pietzsch, T. , Preibisch, S. , Rueden, C. , Saalfeld, S. , Schmid, B. , Tinevez, J. Y. , White, D. J. , Hartenstein, V. , Eliceiri, K. , Tomancak, P. , & Cardona, A. (2012). Fiji: An open‐source platform for biological‐image analysis. Nature Methods, 9(7), 676–682. 10.1038/nmeth.2019 22743772 PMC3855844

[ece311236-bib-0102] Schmidt‐Nielsen, K. (1984). Scaling: Why is animal size so important? Cambridge University Press.

[ece311236-bib-0103] Schofield, R. M. S. , Emmett, K. D. , Niedbala, J. C. , & Nesson, M. H. (2011). Leaf‐cutter ants with worn mandibles cut half as fast, spend twice the energy, and tend to carry instead of cut. Behavioral Ecology and Sociobiology, 65(5), 969–982. 10.1007/s00265-010-1098-6

[ece311236-bib-0104] Schultheiss, P. , Nooten, S. S. , Wang, R. , Wong, M. K. L. , Brassard, F. , & Guénard, B. (2022). The abundance, biomass, and distribution of ants on Earth. Proceedings of the National Academy of Sciences of the United States of America, 119(40), e2201550119. 36122199 10.1073/pnas.2201550119 PMC9546634

[ece311236-bib-0105] Schultz, T. R. , & Meier, R. (1995). A phylogenetic analysis of the fungus‐growing ants (Hymenoptera: Formicidae: Attini) based on morphological characters of the larvae. Systematic Entomology, 20(4), 337–370. 10.1111/j.1365-3113.1995.tb00100.x

[ece311236-bib-0106] Seabold, S. , & Perktold, J. (2010). statsmodels: Econometric and statistical modeling with python. In *9th Python in science conference*.

[ece311236-bib-0107] Shearer, B. M. , Cooke, S. B. , Halenar, L. B. , Reber, S. L. , Plummer, J. E. , Delson, E. , & Tallman, M. (2017). Evaluating causes of error in landmark‐based data collection using scanners. PLoS One, 12(11), e0187452. 10.1371/journal.pone.0187452 29099867 PMC5669428

[ece311236-bib-0108] Silva, L. C. , Camargo, R. S. , Lopes, J. F. S. , & Forti, L. C. (2016). Mandibles of leaf‐cutting ants: Morphology related to food preference. Sociobiology, 63, 881. 10.13102/sociobiology.v63i3.1014

[ece311236-bib-0109] Silva, T. S. R. , & Feitosa, R. M. (2019). On titles and royalty: A terminological discussion over castes in myrmecology. Insectes Sociaux, 66(1), 25–35. 10.1007/s00040-018-0672-1

[ece311236-bib-0110] Tautz, J. , Roces, F. , & Hölldobler, B. (1995). Use of a sound‐based vibratome by leaf‐cutting ants. Science, 267(5194), 84–87. 10.1126/science.267.5194.84 17840064

[ece311236-bib-0111] Tawdros, S. , West, M. , & Purcell, J. (2020). Scaling relationships in Formica ants with continuous worker size variation. Insectes Sociaux, 67(4), 463–472. 10.1007/s00040-020-00779-0

[ece311236-bib-0112] Thompson, D. W. (1917). On growth and form. Cambridge University Press.

[ece311236-bib-0113] Toussaint, N. , Redhead, Y. , Vidal‐García, M. , Lo Vercio, L. , Liu, W. , Fisher, E. M. C. , Hallgrímsson, B. , Tybulewicz, V. L. J. , Schnabel, J. A. , & Green, J. B. A. (2021). A landmark‐free morphometrics pipeline for high‐resolution phenotyping: Application to a mouse model of Down syndrome. Development, 148(18), dev188631. 10.1242/dev.188631 33712441 PMC7969589

[ece311236-bib-0114] Trible, W. , & Kronauer, D. J. (2017). Caste development and evolution in ants: it's all about size. Journal of Experimental Biology, 220(1), 53–62. 28057828 10.1242/jeb.145292

[ece311236-bib-0115] Tschinkel, W. R. (2013). The morphometry of Solenopsis fire ants. PLoS One, 8(11), e79559. 10.1371/journal.pone.0079559 24260250 PMC3834273

[ece311236-bib-0116] Vieira, A. S. , Camargo‐Mathias, M. I. , & Roces, F. (2015). Comparative morpho‐physiology of the metapleural glands of two Atta leaf‐cutting ant queens nesting in clayish and organic soils. Arthropod Structure & Development, 44(5), 444–454. 10.1016/j.asd.2015.06.005 26145506

[ece311236-bib-0117] Villet, M. H. (1992). Definitions of «caste» in social insects. Ethology Ecology & Evolution, 4(3), 213–224. 10.1080/08927014.1992.9523134

[ece311236-bib-0118] von Cramon‐Taubadel, N. , Frazier, B. C. , & Lahr, M. M. (2007). The problem of assessing landmark error in geometric morphometrics: Theory, methods, and modifications. American Journal of Physical Anthropology, 134(1), 24–35. 10.1002/ajpa.20616 17503448

[ece311236-bib-0119] Weber, N. A. (1972). Gardening ants the attines .

[ece311236-bib-0120] Wetterer, J. K. (1991). Allometry and the geometry of leaf‐cutting in Atta cephalotes. Behavioral Ecology and Sociobiology, 29(5), 347–351. 10.1007/BF00165959

[ece311236-bib-0121] Wetterer, J. K. (1995). Forager polymorphism and foraging ecology in the leaf‐cutting ant, Atta colombica. Hindawi Limited. https://doaj.org/article/935e2579d20e4982baf66797e60a4be7 10.1007/BF00341478 28313983

[ece311236-bib-0122] Wetterer, J. K. (1999). The ecology and evolution of worker size‐distribution in leaf‐cutting ants (Hymenoptera: Formicidae). Sociobiology, 34, 119–144.

[ece311236-bib-0123] Wetterer, J. K. (2012). Worldwide spread of the African big‐headed ant, Pheidole megacephala (Hymenoptera: Formicidae). Myrmecological News, 17, 51–62.

[ece311236-bib-0124] Wheeler, D. E. (1986). Developmental and physiological determinants of caste in social Hymenoptera: Evolutionary implications. The American Naturalist, 128(1), 13–34.

[ece311236-bib-0125] Wheeler, D. E. (1991). The developmental basis of worker caste polymorphism in ants. The American Naturalist, 138(5), 1218–1238. 10.1086/285279

[ece311236-bib-0126] Wills, B. D. , Powell, S. , Rivera, M. D. , & Suarez, A. V. (2018). Correlates and consequences of worker polymorphism in ants. Annual Review of Entomology, 63, 575–598. 10.1146/annurev-ento-020117-043357 29068707

[ece311236-bib-0127] Wilson, E. O. (1953). The origin and evolution of polymorphism in ants. The Quarterly Review of Biology, 28(2), 136–156. 10.1086/399512 13074471

[ece311236-bib-0140] Wilson, E. O. (1963). The social biology of ants. Annual Review of Entomology, 8(1), 345–368.

[ece311236-bib-0128] Wilson, E. O. (1968). The ergonomics of caste in the social insects. The American Naturalist, 102(923), 41–66. 10.1086/282522

[ece311236-bib-0129] Wilson, E. O. (1976). A social ethogram of the neotropical arboreal ant Zacryptocerus varians (Fr. Smith). Animal Behaviour, 24(2), 354–363. 10.1016/S0003-3472(76)80043-7

[ece311236-bib-0130] Wilson, E. O. (1979). The evolution of caste systems in social insects. Proceedings of the American Philosophical Society, 123(4), 204–210.

[ece311236-bib-0131] Wilson, E. O. (1980a). Caste and division of labor in leaf‐cutter ants (Hymenoptera: Formicidae: Atta) I. The overall pattern in *A. sexdens* . Behavioral Ecology and Sociobiology, 7(2), 143–156. 10.1007/BF00299520

[ece311236-bib-0132] Wilson, E. O. (1980b). Caste and division of labor in leaf‐cutter ants (Hymenoptera: Formicidae: Atta) II. The ergonomic optimization of leaf cutting. Behavioral Ecology and Sociobiology, 7(2), 157–165. 10.1007/BF00299521

[ece311236-bib-0133] Wilson, E. O. (1983). Caste and division of labor in leaf‐cutter ants (Hymenoptera: Formicidae: Atta) IV. Colony ontogeny of *A. cephalotes* III. Ergonomic resiliency in foraging by *A. cephalotes* . Behavioral Ecology and Sociobiology, 14(1), 55–60. 10.1007/BF00366656

[ece311236-bib-0134] Wilson, E. O. (1984). The relation between caste ratios and division of labor in the ant genus Pheidole (Hymenoptera: Formicidae). Behavioral Ecology and Sociobiology, 16(1), 89–98. 10.1007/BF00293108

[ece311236-bib-0135] Wilson, E. O. (1985). The principles of caste evolution. Fortschritte der Zoologie, 31, 307–324.

[ece311236-bib-0136] Wilson, E. O. (1990). Success and dominance in ecosystems: The case of the social insects. Ecology Institute Oldendorf/Luhe.

[ece311236-bib-0137] Wolf, I. , Vetter, M. , Wegner, I. , Böttger, T. , Nolder, M. , Schöbinger, M. , Hastenteufel, M. , Kunert, T. , & Meinzer, H. (2004). The medical imaging interaction toolkit (MITK): A toolkit facilitating the creation of interactive software by extending VTK and ITK. In Medical imaging 2004: Visualization, image‐guided procedures, and display (pp. 16–27). SPIE.

[ece311236-bib-0138] Yushkevich, P. A. , Piven, J. , Hazlett, H. C. , Smith, R. G. , Ho, S. , Gee, J. C. , & Gerig, G. (2006). User‐guided 3D active contour segmentation of anatomical structures: Significantly improved efficiency and reliability. NeuroImage, 31(3), 1116–1128. 16545965 10.1016/j.neuroimage.2006.01.015

