# Supplementary material for: Large deformation diffeomorphic mapping of 3D shape variation reveals two distinct mandible and head capsule morphs in Atta vollenweideri leaf‐cutter worker ants
Source: Ecol Evol. 2024 Apr 16;14(4):e11236. doi: 10.1002/ece3.11236 (PMC11021802; doi:10.1002/ece3.11236)
Supplement: Supplementary file 1 — Appendix S1 [file ECE3-14-e11236-s001.docx]

**Supporting Information**

**Appendix S1. Details of scanning procedure.**

*Queen Scan*

The queen was scanned at the Bioengineering Core Facilities at Imperial College London, using a Zeiss Xradia 510 Versa 3D X-ray microscope (Carl Zeiss XRM Inc., Dublin, CA, USA). The tube voltage was set at 80 kV, the current to 88 μA and the exposure time to 3000 ms. A total of 6001 projections were captured at 4x lens magnification with 2x binning over a full 360 degrees range. Post-imaging 3D reconstruction from the 2D projections was performed using software package XMReconstructor (Carl Xeiss XRM Inc., Dublin, CA, USA), resulting in 8-bit greyscale image stacks with an isotropic voxel size of 6.48 μm.

*Worker Scans*

The workers were scanned using a Xradia Versa 520 (Carl Zeiss XRM Inc., Dublin, CA, USA) with a tube voltage of 70 kV, a current of 85 μA, a exposure time of 500 ms, a CCD detector system with scintillatorcoupled visible light optics, and a tungsten transmission target. A low energy filter was placed in the beam path (LE1, proprietary Carl Zeiss microscopy filter), and a total of 2401 projections were captured at a 4x lens magnification with 2x binning over a ‘180 degrees plus fan angle’ range. The tomograms were reconstructed from 2D projections using a commercial software package (XMReconstructor, Carl Zeiss XRM Inc., Dublin, CA, USA), with a cone-beam reconstruction algorithm based on filtered back-projection, resulting in 8-bit greyscale image stacks with isotropic voxel sizes between 2.6 to 3.4 μm.


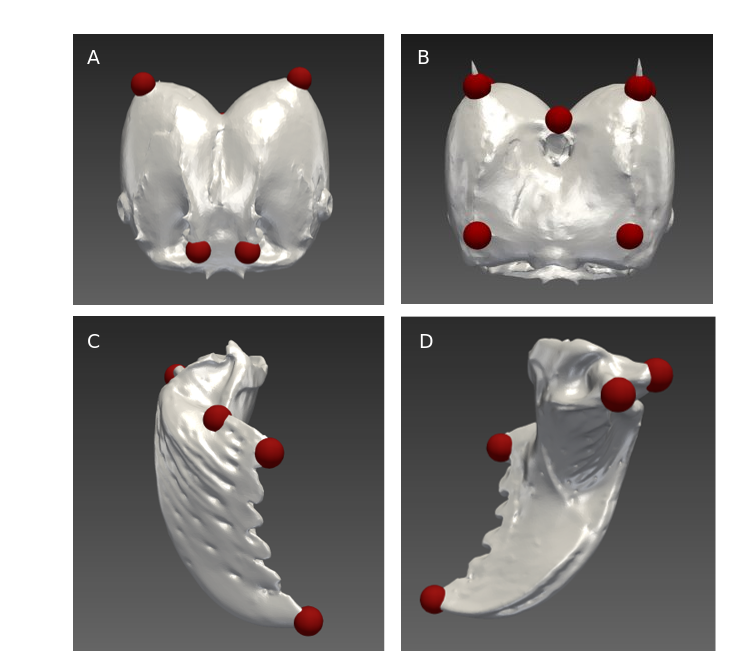


**Figure S1. Points used for initial alignment and scaling step in analysis.** Prior to creation of a shape atlas, all samples were aligned and scaled. We used a landmark-facilitated Procustes alignment to reposition and rescale the mesh files. The landmarks were manually placed using MITK Workbench (red dots in **(A-B)** and **(C-D)** for the head capsules and mandibles, respectively). The head capsules received a total of 9 landmarks, and the mandibles had a total of 5 landmarks (description in Table S1. See main text, Fig. 1C for images of the alignment).

**Table S1.** Location of landmark points used for Procustes alignment, corresponding to the points indicated in Figure S1.

|  | **Head** | **Mandible** |
| --- | --- | --- |
| 1 | left anterior torulus point | apical tooth |
| 2 | right anterior torulus point | basal tooth |
| 3 | left dorsal vertex spine | anterior acetabulum |
| 4 | right dorsal vertex spine | dorsal condyle |
| 5 | left anterior ridge of postgenal bridge | ventral condyle |
| 6 | right anterior ridge postgenal bridge |  |
| 7 | posterior point median above the postoccipital carina |  |
| 8 | right posterior vertex spine |  |
| 9 | left posterior vertex spine |  |

**Table S2.** Definition of anatomical terms used in text, definitions from Richter *et al.* 2019 *Arthropod Structure and Development* and Gauld and Bolton 1988.

| **Term** | **Definition** |
| --- | --- |
| acetabulum | concave depression of torular sclerite receiving the bulbus |
| atala | lateral swelling of mandibular base internally connected to abductor apodeme, also known as "abductor swelling" |
| apical incisor | the bottom-most tooth serving as the tip or cutting edge of the mandible |
| basal margin | mandibular margin connecting masticatory margin and mandibular base |
| basal angle | the angle at which the mandibular margin and masticatory margin meet |
| canthellus | strengthening ridge between basal margin and mandibular base |
| masticatory margin | toothed distal edge of the mandible |
| occipital foramen | posterior opening of the head capsule connecting the head with the digestive tubes |
| postgenal bridge | median fusion of the postgenae, forming a bridge that separates the foramen mangum from the oral fossa |
| postoccipital carina | rim which surrounds the occipital foramen |
| torulus | circular sclerite with annular rim surrounding antennal acetabulum |
| trulleum | concavity of dorsal mandibular base, delimited by basal mandibular margin and canthellus |

**Figure S2.** Comparison of the first 3 principal components to body mass for the (A-C) head capsules and (D-E) mandibles. The relationship between corresponding principal components for the head capsules and mandibles are plotted for (G) PC1, (H) PC2 and (I) PC3. The colour of the points represents mass from dark (small mass) to light (large mass).

N=50

N=50

N=697

**Figure S3.**  To estimate the size-range of workers that tend to cut leaves, forage, or maintain the fungal garden, we counted and weighed ants engaged in each behaviour. To estimate the size of workers that leave the nest to forage, **(A)** 50 workers that were not actively cutting were randomly selected from the foraging container. **(B)** To estimate the number of ants cutting leaves, we randomly removed 50 workers that were actively cutting bramble leaves in the foraging area within an hour of leaves being provided to the colony. **(C)** To estimate the worker size distribution of fungal garden workers, we removed 100 mL of fungus and carefully separated the ants from the fungus. We first visually separated the ants into size groups: greater than 5mg, 1-5mg, and less than 1mg. We then weighed all of the ants in the two largest size classes as these classes contained a smaller number of ants (n=225). However, as the smallest size class contained a large number of ants, and the resolution of the scale prohibited accurate measurements at the smallest end of the range (some ants failed to register on the scale), we only measured 25 ants in the smallest class to assess the accuracy of separating by eye. We found that only one ant (4%) was slightly above 1mg, weighing 1.1mg. We thus counted the remaining ants in the smallest group and binned the data on 1mg intervals, assigning 4% of those counted in the smallest class to the bin of 1-2mg. The histograms reflect this binning with each bar representing a 1mg interval.
